# Fermentation Efficiency and Profile of Volatile Compounds in Rye Grain Mashes from Crops Fertilised with Agrifood Waste Ashes

**DOI:** 10.3390/molecules30153251

**Published:** 2025-08-02

**Authors:** Łukasz Ściubak, Andrzej Baryga, Maria Balcerek, Katarzyna Pielech-Przybylska, Urszula Dziekońska-Kubczak, Stanisław Brzeziński

**Affiliations:** 1 Institute of Agricultural and Food Biotechnology—State Research Institute, Rakowiecka 36, 02-532 Warszawa, Poland; 2 Department of Sugar Industry and Food Safety Management, Faculty of Biotechnology and Food Sciences, Lodz University of Technology, Wolczanska 171/173, 90-530 Lodz, Poland; andrzej.baryga@p.lodz.pl (A.B.); stanislaw.brzezinski@p.lodz.pl (S.B.); 3 Institute of Fermentation Technology and Microbiology, Faculty of Biotechnology and Food Sciences, Lodz University of Technology, Wolczanska 171/173, 90-530 Lodz, Poland; maria.balcerek@p.lodz.pl (M.B.); urszula.dziekonska-kubczak@p.lodz.pl (U.D.-K.)

**Keywords:** biomass ashes, ethanol fermentation, distillery mash, aroma profile

## Abstract

The utilisation of agrifood waste ashes has the potential to enhance the nutrient content of cereal crops, thereby optimising both yield and grain quality. This study investigated rye grain composition, the fermentation efficiency, and volatile compounds in mashes made from crops fertilised with agrifood waste ashes derived from the combustion of corn cob, wood chips, and biomass with defecation lime. The ashes were applied at 2, 4, and 8 t/ha, separately and as mixtures of corn cob (25%) with wood chips (75%) and corn cob (50%) with biomass and defecation lime (50%). Rye mashes were prepared using the pressureless starch liberation method. The starch content in the majority of the rye grains was comparable to the control sample (57.12 g/100 g). The range of ethanol concentrations observed in the fermented mashes was from 55.55 to 68.12 g/L, which corresponded to fermentation yields of 67.25–76.59% of theoretical. The lowest fermentation yield was exhibited by the mash derived from rye cultivated on soil fertilised with a 50:50 mixture of ashes from corn cob and biomass with defecation lime at 8 t/ha. This mash contained more than double the acetaldehyde concentration and total aldehyde content compared to the other samples. These findings demonstrate the potential of using waste biomass ash as a source of macro- and microelements for rye cultivation, enabling the production of agricultural distillates. To ensure high fermentation efficiency and low aldehyde levels, ash dosage and composition need to be established based on experimental optimisation.

## 1. Introduction

The circular economy relies heavily on biomass as a renewable resource that can be processed sustainably multiple times. The use of biomass for energy production has significant environmental advantages, including reducing greenhouse gas emissions and limiting the use of non-renewable energy sources. However, the combustion of biomass results in the formation of solid combustion waste (ash). According to various estimates, between 480 and 500 million t of ash is already generated globally each year [1]. One promising approach, in line with the principles of the circular economy, is to use the ashes produced by biomass combustion in agriculture [2]. The advantages of this approach include improved soil balance, by adding mineral ingredients present in ashes from biomass combustion (mainly C, O, H, Ca, and K, as well as N, S, Mg, P, Cl, Na, Mn, Zn, Fe, B, Cu, Mo, and others), as well as enhanced soil quality, including its texture, aeration, and water retention capacity [3,4,5].

Fertilisation is a crucial aspect of crop cultivation, improving both the size of the crop and the quality of the grain. However, fertiliser costs can be high, especially when using mineral fertilisers. Therefore, ashes from biomass combustion may offer an economically viable alternative [6].

Meller and Bilenda [7] conducted research on the use of biomass ashes to fertilise corn grown for energy purposes. They observed that the use of the ash at a dose of 60 t/ha produced approximately 10% higher corn yields compared to plants fertilised with commonly available mineral fertilisers. Additionally, the application of biomass ash increased the Mg and P content in the corn grain, with the best results obtained for the largest ash doses (60 and 120 t/ha). The same authors noted an increase in K, Ca, and Na content in corn grain compared to the control sample. Increases in the concentrations of Mn, Zn, and Cu were reported in the green mass of corn.

In the study conducted by Bunevičienė et al. [8], the yield of spring barley grain and straw of crops fertilised with bulk and granular biofuel ash was evaluated. In addition, the correlation between the yield and the amount of K_2_O applied by different fertilisation products was investigated. The study revealed that fertilisation products significantly increased the yield of grown spring barley grains and straw. The yield of spring barley exhibited a positive correlation with the proportion of K_2_O content in fertiliser products. In turn, Gupta et al. [9] investigated whether the recycling of household ash as fertiliser could lead to a sustainable improvement in soil fertility, whilst concomitantly minimising the accumulation of toxic elements (As, Cd) in rice grain. The results showed that the utilisation of ash has the potential to reduce the requirement for expensive chemical fertilisers, whilst at the same time increasing rice yield and maintaining grain quality, making farming in Bangladesh more sustainable and productive. Fertilisation of cereal crops with biomass ash may also affect the chemical composition of cereal grains. Pycia et al. [10] investigated how the soil type, alternative soil fertilisation with ash from wood biomass combustion, and the interaction of these factors impacted selected physicochemical and rheological properties of barley starch. The results showed these factors have a significant influence on the amylose content, gelatinisation, and retrogradation process parameters, as well as pasting characteristics parameters and viscoelastic properties. Similarly, Czernicka et al. [11] found that using fertilisation with plant biomass ash as an alternative method can have a beneficial effect on protein quality, especially nutritional value, but not on total protein quantity in barley flour. Grains from plants fertilised with mineral nitrogen compounds had the highest protein content, but a low level of free amino acids.

Rye is one of the most commonly cultivated cereal crops in Poland, owing to its long-standing cultivation tradition and strong adaptation to local soil and climate conditions. Rye grain is widely used both in the food industry and as a raw material for animal feed. The main applications are for the production of rye bread and spirit [12]. Taking into account the economic significance of rye grain in Poland, it is important to know the effect of fertilisation of rye crops on the composition and characteristics of chemical components in rye grain. Buksa et al. [13] examined the effect of integrated and intensive fertilisation on the yield and chemical composition of rye grain. The obtained results showed that the implementation of intensive technology incorporating elevated doses of minerals, such as ammonium nitrate, superphosphate and potassium salt, resulted in a 7.9% increase in grain yield and a 3.7% increase in starch content. The starch exhibited a higher share of amylose and a lower molar mass when compared to grain cultivated through the integrated method. Moreover, grain originating from intensive production contained 0.6% more water-soluble arabinoxylan of a high molar mass, compared to grain obtained through the integrated method.

The chemical composition of rye, especially its starch content, in conjunction with processing and enzymatic treatments, plays an important role in determining fermentation efficiency, ethanol yield, and the quality of spirit distillate [14]. Pielech-Przybylska et al. [15] investigated the effects of different rye cultivars and management intensities on the efficiency of ethanol production, and the quantitative and qualitative composition of volatile compounds in the spirit distillates. The results showed that neither the rye cultivar nor the management intensity exhibited a significantly impact on the starch and protein content of the tested rye grains, and the fermentation efficiency. However, both factors significantly influenced the concentrations of volatile compounds in the obtained spirit distillates.

Given the rich chemical composition of biomass-based ashes, and the absence of specific research examining the impact of biomass ashes on rye (*Secale cereale*) cultivation and the composition of rye grain, further research is recommended to determine whether enriching rye crops with agrifood waste ashes may influence the chemical composition of rye grain and its technological value in the alcoholic fermentation process, as well as the profile of volatile compounds produced by yeast. Due to the lack of detailed research results in this area, our study aimed to evaluate the possibility of utilising agrifood waste ashes as fertilisers in the cultivation of rye, with a particular focus on their impact on the chemical composition of grain and their usefulness in the production of spirits. The scope of research included an assessment of the effects of fertilising rye crops with various agrifood waste ashes on the starch and total reducing sugar contents of the rye grain, the results of alcoholic fermentation, and the qualitative and quantitative composition of volatile compounds in the fermented mashes.

## 2. Results and Discussion

### 2.1. Raw Material Analysis

In the first phase of the study, we examined the impact of applying various types and doses of combustion ash (corn cob, CC; wood chips, WCH; biomass and defecation lime, BDL, and their combinations) as fertilisers on the content of starch and reducing sugars in cultivated rye grains. The results are presented in Table 1. In the control sample (without ash addition), the starch content was 57.12 g/100 g. In the majority of cases, the use of fertilisers did not result in substantial deviations from this value. The highest starch content (59.52 g/100 g) was recorded in plants cultivated on soil fertilised with wood chip ash at a dose of 4 t/ha. However, it should be noted that the differences in starch content relative to this sample were not statistically significant in several of the other fertilisation variants. These included the control sample and samples fertilised with corn cob ash (8 t/ha), wood chip ash (all doses), biomass ash and defecation lime (each dose), a mixture of corn slurry (25%) and wood chips (75%) at a dose of 8 t/ha, as well as a variant comprising corn cob (50%) with biomass and defecation lime (50%) at a dose of 2 t/ha. The lowest starch content (53.03 g/100 g) was observed in grain from the crop fertilised with ash produced from the combustion of a mixture of corn cob (50%) and biomass with defecation lime (50%) at a dose of 8 t/ha.

Reducing sugar content exhibited a range of values, from 0.82 g glucose/100 g of raw material (CC25 + WCH75, 2 t/ha) to 1.15 g/100 g (CC, 4 t/ha). A clear decreasing tendency was observed with increasing ash doses of single-component ashes (CC, WCH, BDL). However, no such relationship was noticed in the case ash mixtures (CC25 + WCH75 and CC50 + BDL50). It is also noteworthy that the reducing sugar content observed in the tested rye was lower in comparison to the control sample. The results indicate a slightly lower content of starch and reducing sugars in crops fertilised with biomass-based ashes compared to the literature data concerning grain from traditional crops cultivated with the use of mineral fertilisers [16]. However, it cannot be ruled out that these differences are the result of differences in varieties and climatic conditions, and not due to the types of fertilisers used.

### 2.2. Results of Analysis of Sweet Mashes

We also assessed the impact of using ash for rye fertilisation on the composition of sweet mash (Table 2). As can be seen, the extract values, including both sugars released during hydrolysis and other non-sugar components, were in most cases comparable or higher than in the control sample (16.87 °Blg). Higher values were obtained in the CC and BDL samples (2 and 8 t/ha), in all WCH doses, and when higher doses of ash mixtures were used. A significantly lower extract content (15.68 °Blg) was obtained only in the sample from grain fertilised with the CC50 + BDL50 mixture at the highest tested dose (8 t/ha). This is directly related to the total sugar and dextrin content in the mash. Both the highest extract value and total sugar were determined in the WCH 4 t/ha sample (17.48 °Blg and 182.50 g glucose/L, respectively). Among the sugars determined after hydrolysis, maltose was present in the largest quantities (33.78–51.10 g/L), followed by glucose (11.75–35.04 g/L) and maltotriose (10.35–16.41 g/L). Small amounts of xylose were also detected. However, it should be noted that starch saccharification was carried out using the SSF method, without a separate saccharification stage. Therefore, the hydrolytic enzymes were also active during the fermentation stage, continuing the hydrolysis of starch. Strąk et al. [17] found that sweet mashes prepared from rye grain contained between 43 and 50 g/L of reducing sugars, along with 119–169 g/L of dextrins, when the PLS method was employed. The initial dry matter content of the mashes was found to have a direct correlation with the glucose, maltose and maltotriose content, which was determined to be approximately 20 g/L, 40 g/L and 3.5 g/L, respectively. In contrast, the content of glucose, maltose and maltotriose was determined to be 35 g/L, 73 g/L and 7.8 g/L, respectively, in our previous paper [16].

### 2.3. Results of Analysis of Fermented Mashes

The mash was fermented using distiller’s yeast. Once the process was complete, the mash was analysed for the content of sugars remaining after fermentation (maltotriose, maltose and glucose), as well as by-products (succinic acid, acetic acid, glycerol) and ethanol. The intake of sugars from the mash was calculated based on the sugar content. The yield of fermentation was calculated on the basis of the total sugars content in sweet mashes and the ethanol content in fermented mashes [18]. The results are presented in Table 3 and Table 4. As can be seen, glucose was almost completely utilised in all samples, ranging from 0.008 to 0.036 g/L after fermentation, except for the CC50 + BDL50 8 t/ha sample in which the concentration was 3.074 g/L. The high concentration of glucose in this sample may indicate incomplete utilisation of sugars by yeast and potential disturbances during alcoholic fermentation. The same pattern was observed in the values obtained for the fermented mash extract, which did not exceed 1 °Blg, except in the case of the sample CC50 + BDL50 8 t/ha, in which the extract content was 1.26 °Blg. The maltotriose content in the tested fermented mashes ranged from 1.16 to 1.63 g/L, with the lowest concentration found in the control sample without ash addition during fertilisation. In the case of maltose, the CC25 + WCH75 4 t/ha sample stood out (2.06 g/L), showing a significantly higher content than all other variants. The lowest maltose concentration (0.48 g/L) was observed in the CC50 + BDL50 4 t/ha variant. The intake of total sugars was very high and exceeded 98% in most trials. Only the CC50 + BDL50, 8 t/ha sample had a significantly lower sugar utilisation rate, but it was still quite high (96.34%).

In terms of fermentation metabolites, all samples had similar levels of succinic acid (approx. 1.1–1.2 g/L), with the exception of the CC 2 t/ha and BDL 8 t/ha samples, which showed significantly higher values (1.28 and 1.25 g/L, respectively). In the case of acetic acid, the significantly highest concentration (0.05 g/L) was recorded in the CC50 + BDL50 4 t/ha sample, and the lowest (0.02 g/L) in the CC25 + WCH75 4 t/ha sample, but neither of these concentrations indicates the development of acetic acid bacteria, which are a potential contaminant. The sources of microbial contamination of distillery mashes are threefold: raw materials, water, and air. Moreover, the appropriate yeast preparation and maintaining the hygiene of distillery equipment are important factors in ensuring efficient and high-quality distillery operations [19]. Glycerol, as the main by-product of yeast metabolism, whose primary function is to protect yeast cells against various environmental stress [20], ranged from 5.78 to 6.89 g/L, with the highest concentrations observed in the CC and WCH (2 t/ha) samples. Its concentrations in the tested rye mashes were similar to those determined in the distillery mashes in our previous studies [16].

Ethanol, which is the most important by-product of ethanol fermentation, was determined in mashes in amounts ranging from 55.55 to 68.12 g/L (Table 3). Based on the concentration of ethanol, the fermentation yield was also calculated (Table 4). The fermentation yield ranged from 67.25 to 76.59% of the theoretical yield, which is in line with the results of our previous studies [18]. When comparing the results obtained with the control sample of mash made from rye fertilised with standard fertilisers, a comparable ethanol yield was observed in all samples except for the sample fertilised with a mixture of CC50 + BDL50 at a dose of 8 t/ha. In this trial, both the ethanol concentration and the fermentation yield were significantly lower than in the control trial and all other trials (55.55 g/L and 67.25% yield, respectively). Comparable or even higher fermentation yields were observed in the other variants. As reported by Balcerek et al. [14], a yield of 61.5 g/L of ethanol was obtained in fermented mash obtained from unmalted rye along with rye malt. The residual sugars, as determined by the authors, were found to be less than 3.5 g/L, with half of these being maltotriose. Furthermore, the dextrin content was determined to be 14.5 g/L. As was demonstrated in our previous paper [16], comparable outcomes were obtained. The ethanol concentration was found to be within the range of 62 to 66 g/L, while the efficiency of the fermentation process varied from 67 to 82%.

### 2.4. Volatile Compounds in the Fermented Mashes

In the second stage of the study, we assessed the quantitative composition of volatile compounds present in the mashes after the completed fermentation process. The purpose was to investigate whether supplementing traditional mineral soil fertilisation with ashes obtained from agrifood industry waste affects the volatile compound concentrations in fermented mash. The reference sample was a mash prepared from rye grown on soil fertilised with traditional mineral fertilisers, ammonium nitrate (AN), 34% N, and ammonium phosphate (AP) 12% N 52% P_2_O_5_.

The profile of volatile compounds that form during the fermentation of distillery mash is crucial to the quality of the agricultural distillate. During fermentation, yeast synthesises numerous volatile compounds, including carbonyl compounds (aldehydes and ketones), higher alcohols, and ethyl and acetate esters, which determine the taste and aroma of spirits. The agricultural distillate is purified in subsequent technological stages, and the resulting agricultural ethyl alcohol can be used in the production of spirits, including vodkas. Agricultural distillate is also used to make so-called natural vodkas, following corrective distillation. Because of their effects on the sensory profile of spirit drinks, it is important that the concentration of volatile compounds is not too high.

**Figure 1:**
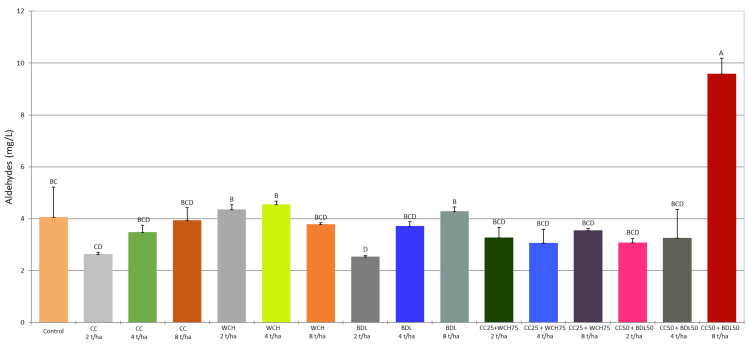
Sum of aldehydes content in fermented mashes. (A–D—different capital letters, indicate significant differences (*p* < 0.05) between the mean values of the aldehydes content, ANOVA).

**Figure 2:**
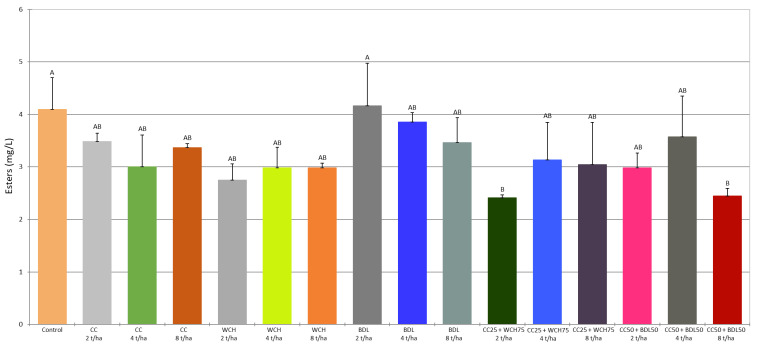
Sum of esters content in fermented mashes. (A,B—different capital letters, indicate significant differences (*p* < 0.05) between the mean values of the esters content, ANOVA).

**Figure 3:**
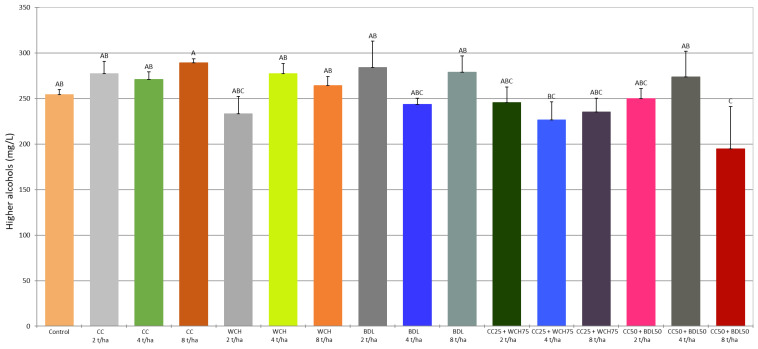
Sum of higher alcohols content in fermented mashes. (A–C—different capital letters, indicate significant differences (*p* < 0.05) between the mean values of the higher alcohols content, ANOVA).

In order to determine the content of volatile compounds, the mash was analysed by gas chromatography (GC) coupled to mass spectrometry (MS), using the headspace (HS) analysis technique. The results for each sample (control and test samples) were subjected to analysis of variance (ANOVA). The statistical significance of the differences between the averages was assessed at a significance level of *p* < 0.05. The results are summarised in Table 5, Table 6 and Table 7 and Figure 1, Figure 2 and Figure 3. The ANOVA analysis showed that most of the volatile compounds in the rye mash made from rye grown on soil fertilised with a mixture of traditional mineral fertilisers and ash occurred at similar levels to the control sample. Only in a few cases were statistically significant differences in concentrations found between the different fertilisation variants and the control. What follows is a comparison of the concentrations of selected volatile compounds in mash obtained from rye grown on soil enriched with agrifood waste ash, taking into account significant differences from the control.

Acetaldehyde (ethanal) is a key carbonyl compound present in spirits. It is strictly regulated in the European Union, particularly in ethyl alcohol of agricultural origin (rectified spirit), due to its toxicity [21]. Since 2022, EU Commission Regulation (EU) 2022/1303 specifies that the acetaldehyde limit includes the combined content of ethanal and 1,1-diethoxyethane (acetal diethyl acetaldehyde) [22]. In our study, the total content of all aldehydes determined in the mashes (ethanal, hexanal, 2-methylpropanal, 2-methylbutanal, 3-methylbutanal and furan-2-carbaldehyde) ranged from 2.52 to 9.57 mg/L (Table 5 and Table 7, Figure 1). The content of acetaldehyde was the highest, ranging from 70.36% to 93.83%. In the control sample, the average ethanal concentration was 3.28 mg/L. Most of the mashes obtained from rye grown on soil fertilised with the tested ashes had comparable acetaldehyde contents to the control sample, ranging from 1.97 mg/L to 3.62 mg/L. In two samples, the acetaldehyde concentration was statistically different from the reference sample. In the BDL 2 t/ha trial, aldehyde levels were significantly lower (1.78 mg/L) than in the control trial, with no statistical differences found for almost any rye trials grown with ash. The exception was the CC50 + BDL50 8 t/ha sample, in which the concentration of acetaldehyde, as well as the concentration of all aldehydes, was more than twice as high as the control sample and the other mashes obtained from rye grown with agrifood waste ash. The content of the other aldehydes including isobutyric aldehyde (2-methylpropanal), 2-methylbutyric aldehyde (2-methylbutanal), and isovaleric aldehyde (3-methylbutanal) in the samples using the ashes ranged from 31.1 to 100.9 μg/L. Similarly to acetaldehyde, the highest concentration of these aldehydes was determined in the mash obtained from rye grown on soil fertilised with conventional mineral fertilisers in combination with a mixture of corn cob ash and from biomass with defecation lime at the highest dose (8 t/ha). Differences were noted in comparison both to the control trial and to most of the mash trials using ash. As previously mentioned, the acetaldehyde limit in ethyl alcohol of agricultural origin is defined as the combined concentration of acetaldehyde and acetaldehyde diethyl acetal (1,1-diethoxyethane). In the quantitative analysis of 1,1-diethoxyethane in the attenuated mash prepared from rye grown on soil fertilised with ashes obtained from CC, WCH, and BDL, no significant differences were found compared to the control (Table 5). However, there were statistically significant differences between some samples in acetal diethyl acetaldehyde concentrations, depending on the dose of ash used for fertilisation. When the ashes from CC, WCH, and BDL were applied individually, higher concentrations of 1,1-diethoxyethane were associated with the higher doses (8 t/ha (CC), 4 and 8 t/ha (WCH) and 8 t/ha (BDL)). No differences were found in the mashes obtained from rye grown with the ash mixture. Furfural (furan-2-carbaldehyde) is also an aldehyde, which, similarly to acetaldehyde, is limited in ethyl alcohol of agricultural origin [22]. Its presence is mainly due to the thermal treatment of starchy raw materials, so the composition of the raw material can significantly affect its concentration in the mash and thus in the final spirits. The concentrations of furfural in the fermented mash samples were low, ranging from 14.7 to 26.6 μg/L, and did not differ significantly from each other (Table 7). The lack of differences suggests that the use of the tested ashes for fertilisation at doses of 2–8 t/ha does not affect furfural formation during heat treatment of rye grains.

Ethyl acetate (ethyl ethanoate) is the most abundant ester produced by yeast during fermentation and is therefore subject to limits in ethyl alcohol of agricultural origin, as specified in EU Commission Regulation 2022/1303 [22]. The content of all esters determined in the mash ranged from 2.41 mg/L to 4.10 mg/L (Figure 2), with ethyl acetate contributing more than 90% of the total esters (Table 5). Ethyl ethanoate is characterised by a fruity aroma, with a solvent-like odour at higher concentrations. Its concentration in the analysed mash remained at a similar level to the control sample (Table 5). However, statistical variation was noticed depending on the type of ash and its dosage. There were differences between the ash variants with BDL (2 t/ha), CC25 + WCH75 (2 t/ha) and CC50 + BDL50 (8 t/ha). In addition to the concentrations of ethyl acetate, concentrations of other compounds from the ester group were also determined in the fermented mash, including ethyl formate (ethyl methanoate), three acetate esters of higher alcohols (isobutyl ethanoate, 2-methylbutyl ethanoate and 3-methylbutyl ethanoate), and eleven fatty acid ethyl esters (ethyl 2-methylbutanoate, ethyl propanoate, ethyl 2-methylpropanoate, ethyl butanoate, ethyl-3methylbutanoate, ethyl pentanoate, ethyl hexanoate, ethyl octanoate, ethyl nonanoate, ethyl decanoate, and ethyl dodecanoate) (Table 5 and Table 6). The level of these compounds qualifies them as minor compounds; however, most have high sensory activity at very low concentrations, and thus play a key role in shaping the aroma profile of spirits.

Lytra et al. [23] conducted a study on aqueous-ethanol model solutions containing 12 different esters, to determine whether compounds present at concentrations below their sensory perception thresholds can influence the overall perception of aroma through synergistic interactions with other compounds from the same chemical group. Some esters, despite very low concentrations, were found to have a significant influence on the intensity of fruit flavours, through synergistic effects. Their co-occurrence in alcoholic beverages can lower the odour perception threshold of the whole mixture and enhance the aromatic sensation. The presence of esters therefore plays a key role in shaping the aroma of alcoholic beverages, primarily contributing to their desirable sensory characteristics.

The level of esters in the tested mash varied from compound to compound. The concentrations of ethyl formate, ethyl propionate, and 3-methylbutyl acetate were in the ranges of 58.0–137.8 μg/L, 29.2–46.8 μg/L, and 16.2–47.7 μg/L, respectively. Other esters such as ethyl isobutyrate, ethyl butyrate, butyl acetate, ethyl isovalerate, ethyl valerate, 2-methylbutyl acetate, ethyl hexanoate, ethyl octanoate, ethyl decanoate, and ethyl dodecanoate were present at concentrations below 10 μg/L and, in the case of ethyl esters of higher fatty acids (≥C6), even below 1 μg/L. Nevertheless, due to the low sensory threshold, even at such low concentrations these esters are classified as highly sensory active compounds. With a few exceptions, no significant differences were noticed between the test samples and the control. For example, in the mash from rye grown with CC ash at 8 t/ha, the concentrations of all ethyl esters of higher fatty acids were higher than the control (Figure 2).

The concentration of higher alcohols in the rye mash was also determined (Table 5 and Table 7). The most common higher alcohols, in quantitative terms, are isoamyl alcohol (3-methylbutan-1-ol), optically active amyl alcohol (2-methylbutan-1-ol), isobutyl alcohol (2-methylpropan-1-ol), and propyl alcohol (propan-1-ol). The content of these compounds, expressed as a sum, ranged from 194.42 mg/L to 288.66 mg/L, with 3-methyl-1-butanol and 2-methyl-1-propanol present in the highest concentrations (Figure 3). The application of the tested ashes did not significantly change the level of 1-propanol compared to the control. Statistically significant differences were observed in the case of isobutyl alcohol. In the mash sample made from rye cultivated with a mixture of ashes with CC and BDL (at a dose of 8 t/ha), the concentration of 1-propanol was lower (by 40%) than the control. The other ash fertilisation variants gave isobutanol concentrations similar to the control.

To support the interpretation of the quantitative analysis of volatile compounds in the attenuated mash, a correlation analysis was performed between the concentrations of volatiles (including ethanol) (Table 3, Table 5, Table 6 and Table 7) and the elemental composition of the rye grains used for mash preparation (Table S1). The elements analysed included N, K, P, Na, Mg, Ca, Mn, Cu, Zn, Fe, Pb, and Cd (Figures S1 and S2). The presence of these metals in the fermentation medium influences yeast metabolism and biochemical processes during fermentation, which can affect fermentation performance and indirectly the volatile compound profile [24,25,26,27,28].

Magnesium is an important enzyme cofactor and regulator of yeast metabolism. It is responsible for prolonging the growth phase of yeast, increasing fermentative activity, and improving ethanol and temperature stress tolerance, resulting in more efficient fermentation and a more favourable secondary metabolite profile [24,25]. Magnesium acts as a cofactor for many enzymes involved in glycolysis and pyruvate metabolism, such as hexokinase, aldolase, glycerophosphate mutase, enolase, and pyruvate kinase. The enzymes of the pyruvate dehydrogenase complex and acetyl-coenzyme A synthetase also require the presence of Mg^2+^ (or Mn^2+^). Magnesium stabilises ribosomal and membrane structures and also protects them from ethanol stress, improving overall fermentation efficiency [24,26].

Other micronutrients involved in yeast metabolism, in addition to magnesium, include manganese, iron, and zinc. Zinc is one of the most important micronutrients for yeast used in ethanol fermentation. It is an essential cofactor of alcohol dehydrogenase (ADH), which catalyses the reduction of acetaldehyde to ethanol [29]. Without sufficient zinc, ADH activity decreases, leading to aldehyde accumulation and slower fermentation. Zinc also activates other enzymes, promoting protein synthesis and amino acid metabolism, and enhances yeast tolerance to stressors, which is important under ethanol fermentation conditions [30]. Copper, while also a necessary trace element, plays a dual role, acting as an enzyme cofactor at low concentrations but having a toxic effect in high concentrations. In excess, copper ions readily generate reactive oxygen species and bind to the thiol groups of proteins, damaging yeast cell structures [27]. Heavy metals, such as lead and cadmium, meanwhile, have no positive biological functions in yeast cells, causing toxic effects even at low concentrations, inducing oxidative stress and inhibiting key enzymatic processes [27].

The zinc content determined of the rye grains used in the present study varied between 17.1 and 23.6 mg/kg with the majority of samples falling below 20 mg/kg (Table S1). In the present study, the determined zinc level was found to be lower in comparison to the data obtained by Klikocka et al. [31] and Ikram et al. [32], i.e., from 24 to 44 mg/kg. No clear effect of the type and dose of ash tested on the level of this element was observed. There was also no significant correlation between zinc and the volatile compounds and ethanol determined (Figure S1).

In the correlation tests (Figures S1 and S2), a moderate to strong correlation was found between the magnesium content of rye grains and the concentrations of some volatile compounds, such as ethanal (r = 0.781, *p* = 0.000), propan-1-ol (r = 0. 628, *p* = 0.009), 2-methylbutan-1-ol (r = −0.614, *p* = 0.011), butan-1-ol (r = −0.519, *p* = 0.039), 2-methylpropan-1-ol (r = −0.595, *p* = 0. 015), 2-methylpropanal (r = 0.620, *p* = 0.011), 2-methylbutanal (r = 0.720, *p* = 0.000), and 3-methylbutanal (r = 0.620, *p* = 0.010). Ribeiro-Filho et al. [26] showed that magnesium influences the fermentation process and the quantitative composition of volatiles. However, the authors observed that its effects may vary depending on the yeast strain. In their study, magnesium affected both the increase and decrease in ethanol, higher alcohols, esters, and acetic acid, depending on the yeast strain. The association of magnesium with the production of acetyl-coenzyme A—a key compound in the biosynthesis of acids, which are precursors of esters—suggests that the presence of magnesium may condition the formation of volatile compounds from the ester group. In our study, we found no correlation between magnesium concentration and the quantitative composition of esters. However, a moderate to strong correlation was observed for aldehydes and higher alcohols. The positive correlation between aldehydes and magnesium in our study contrasts with the findings of Tucillo et al. [28], who reported a reduction in aldehyde concentration. The acetaldehyde concentration in fermented rye mash was also positively correlated with potassium (r = 0.806, *p* = 0.000), sodium (r = 0.646, *p* = 0.007), calcium (r = 0.638, *p* = 0.008), and nitrogen (r = 0.624, *p* = 0.010). An analogous relationship was noted for the other labelled aldehydes—i.e., 2-methylpropanal, 2-methylbutanal, and 3-methylbutanal. This positive correlation makes sense in light of the fact that a negative correlation was noted between ethanol concentration and Mg, N, Ca, K, and Na, as well as between ethanol and the aforementioned aldehydes (Figure S1).

The content of Na, K, Ca and Mg was found to be up to twice as high in rye grains grown in soil fertilised with the CC + BDL ash mixture at the highest dose of 8 t/ha (0.30 g of Na/kg, 8.70 g of K/kg, 0.65 g of Ca/kg, and 0.99 g of Mg/kg) when compared to the other fertilisation variants, including the control trial (Table S1). The ethanol concentration in the mash samples prepared from this grain sample was the lowest (Table 3), while the acetaldehyde concentration was the highest compared to the other fermentation variants (Table 5). Potassium, sodium, calcium and magnesium ions act as osmoregulators and stabilisers of cell membranes [24,33,34]. However, an excess of potassium and calcium ions in the fermentation medium can reduce the yeast fermentation activity and negatively affect yeast growth, as shown in their study by Silva et al. [35]. The cited studies also showed that abnormal proportions of these ions can disrupt cell membrane integrity and lead to ionic stress. Mikulski et al. [36], on the other hand, emphasises the beneficial effects of elements such as Mg and Ca on ethanol yield and cell viability. However, they also point out that maintaining the correct proportions of these two elements it is also important, because too high levels of calcium ions can inhibit yeast cell activity due to the existing antagonism of magnesium and calcium ions. The optimum concentration of these macroelements is a compromise between their osmoregulatory function and the avoidance of ionic stress. In view of the results obtained in this study, the use of ashes obtained from the combustion of corn cob, wood chips and biomass with defecation lime does not interfere with the fermentation process. However, it is important to select the optimum dose of these ashes.

Our correlation analysis revealed a negative relationship between magnesium content and the concentration of three out of four higher alcohols. This differs from the findings of Niu et al. [37], who reported increased levels of higher alcohols in fermentation samples with elevated magnesium content. Tucillo et al. [28], in their study, showed that magnesium indirectly affects acetic acid production, noting that acetic acid levels increased under conditions of prolonged yeast metabolic activity resulting from the presence of magnesium. In our study, the correlation between acetic acid and the elements determined in rye grains was weak (Figure S1).

In our study, no significant correlations were observed between cadmium and lead contents in the rye grains and the concentrations of the determined volatile compounds.

The application of ash did not cause significant changes in the profiles of volatile compounds in fermented rye mash compared to fertilisation with traditional mineral fertilisers (control). The statistically significant differences observed were only related to some of the compounds and were mainly associated with the variant using a mixture of corn cob ash and biomass with defecation lime at the highest dose of 8 t/ha. From the perspective of fermentation and distillation technology, this means that novel, environmentally friendly mineral fertilisers in the form of ashes obtained from the incineration of agrifood waste have potential for application in the cultivation of cereals, including rye, without compromising the quality of the distillates obtained from them.

## 3. Materials and Methods

### 3.1. Materials

The following enzyme preparations (Novozymes, Bagsværd, Denmark) were used during the preparation of mashes:

- Liquoflow—a liquefying preparation (thermostable α-amylase), applied at 0.2 mL/kg of starch;
- Saczyme^®^ Plus—a saccharifying preparation (amyloglucosidase), applied at 0.6 mL/kg of raw material;
- Viscoferm—a mash viscosity-lowering auxiliary preparation (non-starch polysaccharide hydrolases: xylanase, β-glucanase and cellulase), applied at 0.15 mL/kg of raw material;
- Alphalase AFP—an auxiliary preparation (protease) catalysing the hydrolysis of native proteins present in raw materials, increasing the content of free amino nitrogen assimilable by yeast, applied at 1 mL/kg of raw material.

The distillery yeast strain SafSpirit HG-1 (*Saccharomyces cerevisiae*) (Fermentis Division of S.I. Lesaffre, Marcq-en-Baroeul Cedex, France) was applied at a dose of 0.5 g/L mash to start alcoholic fermentation of the prepared mashes.

### 3.2. Rye Cultivation

Rye cultivation experiments were conducted at the Department of Agronomy, Poznań University of Life Sciences (Poland, 52.4328° N, 16.90060° E). Beetroot was sown as a pre-crop in the first year, while rye was grown in the second year. Breeding was conducted in pots placed in the garden. Before the experiment, 7 kg of soil was poured into each pot. The soil in the control and other test trials was fertilised with traditional mineral fertilisers: ammonium nitrate 34% N (AN) and ammonium phosphate 12% N 52% P_2_O_5_ (AP). In addition, ashes from the combustion of corn cob (CC), wood chips (WCH), and biomass in the presence of defecation lime (BDL) were added to the test samples, according to the scheme given in Table 1. The cultures were carried out for two years. In the first year (in spring), 1 g of ammonium nitrate 34% N (AN) and 1 g of ammonium phosphate 12% N 52% P_2_O_5_ (AP) were added to each pot. Ashes were then added (except in the control sample), according to the doses presented in Table 8, before sowing beetroot. In autumn, after harvesting the beetroots, rye was sown using 5 seeds of winter rye from the KWS IGOR cultivar (KWS Polska Sp. z o.o., Poznań, Poland) in each pot. Selection was carried out the following year, in spring, and 3 plants were left in each pot. Nitrogen fertiliser was also applied at 2 g of AN per pot. The plants were kept in the pots until full maturity, at which point they were harvested. The crop experiment was conducted in 5 replicates. Grain samples were used for analysis and fermentation trials.

### 3.3. Course of Mashing and Fermentation Experiments

The rye grains collected from five pots of each trial were mixed and used to prepare three independent samples of sweet mash. The pressureless starch liberation (PLS) method was applied to prepare the rye mashes. The mashing process was conducted in glass beakers placed on a magnetic stirrer equipped with a heating function. A magnetic dipole was placed in each beaker to mix the slurry during the mashing process. Additionally, the temperature sensor was immersed to monitor and regulate the temperature. During the mashing process the beaker was covered with aluminium foil to prevent water evaporation. The previously crushed raw material (20 g) was mixed with tap water in a ratio of 3.5 litres per kilogram of raw material. The mixture was then heated to 65 °C and the enzyme preparation Viscoferm was added to reduce the viscosity of the mash. After reaching a temperature of about 80 °C, the liquefying preparation Liquoflow was added, and the mixture was heated further to 90 °C. At this temperature, the liquefaction process (i.e., starch dextrinization) was continued for 60 min. After this time, the mash was cooled to 65 °C, and the enzymes Saczyme^®^ Plus (amyloglucosidase) and Alphalase AFP (protease) were added. Subsequently, the mash was cooled to about 30 °C, which is a temperature suitable for yeast inoculation. Before fermentation, the pH of the mash was adjusted to 4.8 using 25% (*w*/*v*) sulfuric acid solution. The prepared mash was measured out (100 mL) into a fermentation glass flask (250 mL).

An appropriate amount of yeast was suspended in warm (35 °C) tap water, producing yeast cream. Then, an acid bath was performed by lowering the pH of the suspension to 2 with using 25% (*w*/*v*) sulfuric acid solution and allowing it to stand for 15 min. The treated sample was then added directly to the mash samples. The contents of the flasks were thoroughly mixed, closed with fermentation tubes with glycerine, and placed in a room at a temperature of 32 °C. Fermentation was carried out for 72 h.

### 3.4. Methods

To assess the quality of the raw material, the rye grain was assayed for moisture using a Radwag WPS-30S Moisture Analyser (Radwag, Radom, Poland), as well as for the content of phosphorus, nitrogen, and microelements [38]. The content of reducing sugars and total sugars (after acid hydrolysis) was assayed by the HPLC technique, using an Infinity 1260 liquid chromatograph (Agilent Technologies, Santa Clara, CA, USA) equipped with a refractometer detector (RID), according to the method described by Dziekońska-Kubczak et al. [39]. Based on the results of HPLC analysis, the concentrations of reducing sugars and total sugars were calculated using conversion coefficients to glucose (maltose × 1.05; maltotriose × 1.07) and expressed in g glucose/100 g rye grain. Starch content was calculated as the difference between total sugars and reducing sugars, taking into account the conversion coefficient into starch (0.9), and also expressed in g/100 g rye grain [15].

In sweet mashes, the following parameters were measured: pH (using a HandyLab pH metre, SI Analytics—a Xylem brand, Mainz, Germany), extract content (using the refractometric method, Atago, Tokyo, Japan), and the concentrations of reducing sugars (maltotriose, maltose, glucose) and dextrins (using the HPLC method). In fermented mashes, the content of unfermented sugars, ethanol, and fermentation by-products (succinic acid, lactic acid, acetic acid and glycerol) was determined using the HPLC method [39]. Based on the HPLC results, the fermentation indices (i.e., sugar utilisation and fermentation yield) were calculated [18].

Gas chromatographic analysis (HS–GC/MS) of the volatile compounds in the fermented mashes was performed using a GC apparatus (Agilent 7890A, Agilent Technologies, Santa Clara, CA, USA) coupled to a mass spectrometer (Agilent MSD 5975C, Agilent Technologies, Santa Clara, CA, USA) [15]. The selection of compounds for quantitative analysis was made on the basis of a preliminary qualitative analysis of volatile compounds in fermented mashes carried out in full scan mode. The volatile compounds present in fermented mashes were identified through a comparison of their mass spectra with those stored in the NIST/EPA/NIH Mass Spectra Library (2012; Version 2.0g.) as well as with mass spectra of GC standards. The quantification of the volatile compounds was performed in the selected ion monitoring (SIM) mode using an external calibration method. Calibration solutions containing different concentrations of each GC standard were prepared with 4-heptanone (1 mg/L) used as an internal standard to monitor instrument response and retention time stability. Qualitative and quantitative analysis was performed using Agilent MassHunter B.07.00 software (Agilent Technologies, Santa Clara, CA, USA). All analysis were performed in triplicate. The results were expressed in mg/L or in μg/L.

A capillary column was used to separate volatile compounds (VF-WaxMS, Agilent, USA; 60 m × 0.32 mm × 0.50 μm). The GC oven temperature was programmed to increase from 30 °C (6 min) to 220 °C at a rate of 10 °C/min, where it was maintained for 5 min. The flow rate of the carrier gas (helium) through the column was 1.1 mL/min. The temperature of the injector (split/splitless) was 250 °C. Injections of the tested samples were made in the split mode (25:1) using a headspace analyser (Agilent 7697A, Agilent Technologies, Santa Clara, CA, USA). The temperatures of the MS ion source, transfer line, and quadrupole were 230 °C, 250 °C, and 150 °C, respectively. The ionisation energy was 70 eV. Before analysis, a 20 mL headspace vial was filled with 7 mL of mash and closed tightly using an aluminium cap and septa. Headspace conditions were as follows:

- Temperature settings: oven temperature 50 °C, loop temperature 60 °C, and transfer line temperature 70 °C;
- Timing settings: vial equilibration time 20 min, injection duration 0.7 min, and GC cycle time 47 min;
- Vial and loop settings: vial shaking 71 shakes/min, fill pressure 15 psi, and vial pressurisation gas helium.

### 3.5. Statistical Analysis

All experiments were performed in triplicate. To assess whether there were significant differences in the compositions of the rye grains (starch), mashes (sweet and fermented), and fermentation indices, statistical analysis of the results obtained was performed using a one-way ANOVA with a significance level of 0.05. The correlation between the qualitative composition of volatile compounds and the macro- and microelements determined in the rye grains was also assessed. The following ranges for the Pearson’s correlation coefficient (r) were used to interpret the strength of correlations [40,41,42]:

- |r| = 0.00—no correlation;
- 0 < |r| < 0.3—very weak correlation;
- 0.3 ≤ |r| < 0.5—weak correlation;
- 0.5 ≤ |r| < 0.7—moderate correlation;
- 0.7 ≤ |r| < 0.9—strong correlation;
- 0.9 ≤ |r| ≤ 1.0—very strong correlation.

All statistical analyses were carried out using XLSTAT software 2024.1.0 (Lumivero, Denver, CO, USA).

## 4. Conclusions

The purpose of this study was to investigate the effect of fertilising the soil under rye crops with ashes obtained from the combustion of various agrifood industry wastes (corn cob, wood chips, and biomass and defecation lime) on the chemical composition of the grain and its suitability for distilling purposes. It was found that supplementing traditional mineral fertilisation of the soil with ashes obtained from the incineration of agrifood industry waste did not significantly affect the starch content of most rye samples in relation to the control (grain from a crop fertilised with mineral fertilisers only). There were also no significant differences in fermentation yields or the qualitative and quantitative compositions of volatile compounds in the attenuated mashes. The exception was a mash sample made from grain fertilised with 50% corn cob ash and 50% biomass and defecation lime (CC50 + BDL50, dose 8 t/ha). This sample had the lowest fermentation yield (67.25%) and more than twice the concentration of acetaldehydes compared to the control sample and the other mashes obtained from rye grown with biomass ash. Further studies should be conducted to exclude or confirm the presence of factors inhibiting fermentation of rye grain from crops fertilised with the above-mentioned ashes (CC50 + BDL50, 8 t/ha).

The results of this study support the potential application of waste biomass ash as a source of macro- and microelements necessary for the proper growth of cereals, such as rye, which can then be used for the efficient and sustainable production of agricultural distillates, in line with the principles of the circular economy.

## Figures and Tables

**Table 1:** Sugar content in rye grains cultivated with use of different ashes as fertilisers.

Sample	Total Reducing Sugars g Glucose/100 g		Starch g/100 g	
	Mean	SD	Mean	SD
Control	1.22 ^ab^	0.10	57.12 ^abc^	1.52
CC 2 t/ha	0.87 ^ef^	0.07	55.90 ^bcd^	1.04
CC 4 t/ha	1.15 ^abc^	0.04	55.20 ^bcd^	1.63
CC 8 t/ha	1.29 ^a^	0.16	56.34 ^abcd^	3.21
WCH 2 t/ha	0.96 ^def^	0.04	57.83 ^ab^	2.46
WCH 4 t/ha	1.11 ^bcd^	0.15	59.52 ^a^	0.55
WCH 8 t/ha	1.02 ^cde^	0.07	58.41 ^ab^	1.89
BDL 2 t/ha	0.93 ^ef^	0.09	58.51 ^ab^	2.09
BDL 4 t/ha	0.86 ^ef^	0.01	56.90 ^abc^	1.40
BDL 8 t/ha	1.01 ^cde^	0.09	57.09 ^abc^	0.55
CC25 + WCH75 2 t/ha	0.82 ^f^	0.07	54.12 ^cd^	3.00
CC25 + WCH75 4 t/ha	1.11 ^bcd^	0.09	55.57 ^bcd^	0.44
CC25 + WCH75 8 t/ha	0.88 ^ef^	0.02	58.01 ^ab^	1.03
CC50 + BDL50 2 t/ha	1.11 ^bcd^	0.03	56.81 ^abc^	0.49
CC50 + BDL50 4 t/ha	0.94 ^ef^	0.02	55.64 ^bcd^	1.07
CC50 + BDL50 8 t/ha	0.96 ^def^	0.01	53.03 ^d^	1.71
*p*-values signification codes ^1^	***		***	

^1^ Signification codes: 0 < *** < 0.001 < ** < 0.01 < * < 0.05 < . < 0.1 < ° < 1. ^a–f^—mean values in columns denoted by different letters are significantly different (one-way ANOVA). SD—standard deviation.

**Table 2:** Chemical composition and extract of sweet mashes obtained from rye cultivated with use of different ashes as fertilisers.

Compound Parameter	Control	CC, t/ha			WCH, t/ha			BDL, t/ha			CC25 + WCH75, t/ha			CC50 + BDL50, t/ha			*p*-Values Signification Codes ^1^
		2	4	8	2	4	8	2	4	8	2	4	8	2	4	8	
Maltotriose, g/L	15.627 ^a^	14.836 ^abc^	12.353 ^bcd^	15.598 ^a^	13.611 ^abc^	16.271 ^a^	11.808 ^cd^	16.193 ^a^	13.761 ^abc^	14.047 ^abc^	12.250 ^bcd^	14.037 ^abc^	14.980 ^ab^	10.354 ^d^	16.408 ^a^	15.059 ^ab^	***
SD	1.312	0.217	0.410	1.262	0.706	1.179	1.785	1.774	2.820	0.296	2.471	1.295	1.020	2.465	0.452	0.655	
Maltose, g/L	42.748 ^abcd^	42.490 ^abcd^	46.295 ^abc^	42.015 ^abcd^	47.439 ^a^	41.711 ^abcd^	34.516 ^cd^	48.280 ^a^	50.800 ^a^	51.103 ^a^	35.481 ^bcd^	47.219 ^ab^	48.414 ^a^	33.781 ^d^	44.323 ^abcd^	43.548 ^abcd^	***
SD	0.739	0.135	11.068	4.518	3.993	5.567	8.032	6.619	6.080	10.823	3.171	1.301	0.965	7.908	3.426	1.333	
Glucose, g/L	16.204 ^de^	12.499 ^e^	19.603 ^bcde^	11.749 ^e^	20.347 ^bcde^	18.100 ^cde^	25.701 ^bc^	19.421 ^bcde^	35.040 ^a^	17.097 ^cde^	21.674 ^bcd^	14.569 ^de^	27.954 ^ab^	15.009 ^de^	15.100 ^de^	19.648 ^bcde^	***
SD	5.356	2.496	3.346	1.691	5.350	3.436	3.714	5.376	5.579	3.728	4.702	2.833	5.347	3.959	5.369	1.951	
Total reducing sugars, g glucose/L	1.526 ^cde^	1.462 ^de^	1.702 ^bcde^	1.218 ^e^	1.948 ^bcd^	1.210 ^e^	2.120 ^abc^	1.793 ^bcde^	2.644 ^a^	1.737 ^bcde^	1.827 ^bcde^	1.416 ^de^	2.315 ^ab^	1.278 ^e^	1.594 ^cde^	1.841 ^bcde^	***
SD	0.383	0.149	0.286	0.066	0.357	0.207	0.432	0.347	0.341	0.249	0.442	0.217	0.370	0.278	0.352	0.216	
Total sugars, g glucose/L	78.005 ^cd^	73.178 ^cd^	81.617 ^bc^	72.747 ^cd^	84.918 ^bc^	79.503 ^bc^	74.730 ^cd^	87.654 ^abc^	103.310 ^a^	85.995 ^bc^	72.195 ^cd^	79.367 ^bc^	95.024 ^ab^	61.702 ^d^	79.400 ^bc^	81.680 ^bc^	***
SD	6.666	2.684	13.550	6.757	7.487	4.031	11.066	7.794	6.443	9.402	10.538	2.903	5.401	13.565	6.545	2.035	
Dextrins, g/L	174.835 ^abc^	170.210 ^bcd^	168.877 ^bcd^	174.135 ^abc^	176.250 ^ab^	182.500 ^a^	178.156 ^ab^	178.240 ^ab^	173.196 ^abc^	174.196 ^abc^	164.746 ^cd^	169.896 ^bcd^	176.596 ^ab^	173.613 ^abc^	169.613 ^bcd^	161.858 ^d^	***
SD	4.805	3.047	4.933	7.386	7.283	9.209	5.840	6.380	4.247	1.787	8.825	1.177	3.156	1.409	3.174	5.143	
Extract, °Blg	16.87 ^bcd^	17.33 ^abc^	16.83 ^bcd^	17.72 ^a^	17.37 ^abc^	17.48 ^ab^	17.37 ^abc^	17.37 ^abc^	16.80 ^bcd^	17.38 ^abc^	16.63 ^cd^	17.03 ^abcd^	17.08 ^abcd^	16.53 ^d^	17.37 ^abc^	15.68 ^e^	***
SD	0.79	0.25	0.51	0.41	0.38	0.58	0.33	0.15	0.20	0.12	0.34	0.15	0.04	0.14	0.29	0.50	

^1^ Signification codes: 0 < *** < 0.001 < ** < 0.01 < * < 0.05 < . < 0.1 < ° < 1. ^a–e^—mean values in rows denoted by different letters are significantly different (one-way ANOVA). SD—standard deviation.

**Table 3:** Chemical composition of fermented mashes obtained from rye cultivated with use of different ashes as fertilisers.

Compound	Control	CC, t/ha			WCH, t/ha			BDL, t/ha			CC25 + WCH75, t/ha			CC50 + BDL50, t/ha			*p*-Values Signification Codes ^1^
		2	4	8	2	4	8	2	4	8	2	4	8	2	4	8	
Maltotriose, g/L	1.163 ^j^	1.568 ^abc^	1.546 ^abcd^	1.628 ^a^	1.407 ^defg^	1.524 ^abcde^	1.464 ^bcde^	1.262 ^hij^	1.276 ^ghij^	1.595 ^ab^	1.479 ^bcde^	1.398 ^efgh^	1.160 ^j^	1.445 ^cdef^	1.320 ^fghi^	1.182 ^ij^	***
SD	0.005	0.013	0.007	0.007	0.004	0.001	0.002	0.005	0.007	0.005	0.004	0.004	0.003	0.004	0.003	0.000	
Maltose, g/L	0.875 ^ef^	1.091 ^cde^	1.357 ^bcd^	0.797 ^ef^	1.458 ^bc^	0.948 ^de^	0.989 ^de^	0.823 ^ef^	1.028 ^de^	1.122 ^cde^	1.103 ^cde^	2.064 ^a^	0.955 ^de^	0.907 ^e^	0.478 ^f^	1.569 ^b^	***
SD	0.026	0.114	0.053	0.004	0.076	0.012	0.012	0.010	0.027	0.003	0.002	0.001	0.000	0.045	0.107	0.165	
Glucose, g/L	0.021 ^b^	0.012 ^b^	0.015 ^b^	0.008 ^b^	0.009 ^b^	0.013 ^b^	0.022 ^b^	0.022 ^b^	0.020 ^b^	0.014 ^b^	0.013 ^b^	0.010 ^b^	0.020 ^b^	0.016 ^b^	0.036 ^b^	3.074 ^a^	***
SD	0.000	0.000	0.000	0.000	0.000	0.000	0.000	0.000	0.000	0.000	0.000	0.000	0.000	0.000	0.001	5.618	
Xylose, g/L	0.584 ^b^	0.741 ^b^	0.564 ^b^	0.585 ^b^	0.420 ^b^	0.557 ^b^	0.610 ^b^	0.605 ^b^	0.584 ^b^	0.651 ^b^	0.611 ^b^	0.689 ^b^	0.578 ^b^	0.619 ^b^	0.435 ^b^	1.798 ^a^	***
SD	0.001	0.009	0.000	0.000	0.043	0.000	0.002	0.001	0.001	0.002	0.001	0.002	0.000	0.001	0.041	1.045	
Succinic acid, g/L	1.129 ^cd^	1.280 ^a^	1.141 ^bcd^	1.193 ^abc^	1.073 ^d^	1.187 ^abc^	1.166 ^bcd^	1.153 ^bcd^	1.162 ^bcd^	1.245 ^ab^	1.171 ^bcd^	1.179 ^abcd^	1.156 ^bcd^	1.129 ^cd^	1.130 ^cd^	1.171 ^bcd^	***
SD	0.004	0.001	0.001	0.001	0.007	0.001	0.001	0.006	0.001	0.000	0.000	0.001	0.001	0.000	0.015	0.004	
Glycerol, g/L	6.003 ^ef^	6.887 ^a^	6.197 ^cdef^	6.382 ^bcde^	6.868 ^a^	6.334 ^bcde^	6.344 ^bcde^	5.784 ^f^	6.463 ^abcd^	6.683 ^ab^	6.293 ^bcde^	6.485 ^abc^	6.172 ^cdef^	6.099 ^cdef^	6.075 ^cdef^	6.022 ^def^	***
SD	0.119	0.085	0.025	0.013	0.153	0.023	0.038	0.132	0.005	0.002	0.019	0.010	0.021	0.039	0.007	0.104	
Acetic acid, g/L	0.041 ^ab^	0.021 ^cde^	0.034 ^abcde^	0.034 ^abcde^	0.038 ^abc^	0.020 ^de^	0.035 ^abcd^	0.028 ^abcde^	0.039 ^ab^	0.032 ^abcde^	0.028 ^abcde^	0.017 ^e^	0.030 ^abcde^	0.027 ^bcde^	0.046 ^a^	0.032 ^abcde^	***
SD	0.000	0.000	0.000	0.000	0.000	0.000	0.000	0.000	0.000	0.000	0.000	0.000	0.000	0.000	0.001	0.000	
Ethyl alcohol, g/L	64.287 ^ab^	66.041 ^ab^	65.354 ^ab^	68.122 ^a^	65.706 ^ab^	67.065 ^ab^	67.563 ^a^	63.327 ^b^	66.925 ^ab^	67.720 ^a^	63.048 ^b^	64.459 ^ab^	64.708 ^ab^	63.091 ^b^	65.920 ^ab^	55.549 ^c^	***
SD	14.376	3.643	6.429	3.082	1.969	7.235	0.464	8.960	0.879	0.738	1.564	0.647	1.281	0.800	8.463	2.171	
Total sugars, g glucose/L	2.192 ^c^	2.847 ^bc^	3.106 ^bc^	2.597 ^bc^	3.057 ^bc^	2.649 ^bc^	2.637 ^bc^	2.245 ^bc^	2.473 ^bc^	2.911 ^bc^	2.765 ^bc^	3.686 ^b^	2.273 ^bc^	2.524 ^bc^	1.958 ^c^	5.996 ^a^	***
SD	0.049	0.064	0.072	0.023	0.062	0.011	0.023	0.024	0.068	0.016	0.000	0.001	0.002	0.024	0.068	7.707	

^1^ Signification codes: 0 < *** < 0.001 < ** < 0.01 < * < 0.05 < . < 0.1 < ° < 1. ^a–j^—mean values in rows denoted by different letters are significantly different (one-way ANOVA). SD—standard deviation.

**Table 4:** Parameters of fermented mashes obtained from rye cultivated with use of different ashes as fertilisers and indices of fermentation.

Parameter/Indices	Control	CC, t/ha			WCH, t/ha			BDL, t/ha			CC25 + WCH75, t/ha			CC50 + BDL50, t/ha			*p*-Values Signification Codes ^1^
		2	4	8	2	4	2	4	8	2	4	2	4	8	2	4	
Extract, °Blg	0.65 ^b^	0.67 ^b^	0.82 ^b^	0.69 ^b^	0.89 ^b^	0.78 ^b^	0.81 ^b^	0.63 ^b^	0.66 ^b^	0.77 ^b^	0.91 ^b^	0.81 ^b^	0.72 ^b^	0.67 ^b^	0.81 ^b^	1.26 ^a^	***
SD	0.05	0.16	0.07	0.01	0.04	0.02	0.07	0.05	0.04	0.05	0.07	0.06	0.04	0.08	0.09	0.52	
pH	3.88 ^gh^	4.14 ^abcd^	4.13 ^bcd^	4.06 ^cdef^	4.30 ^a^	4.04 ^cdefg^	4.09 ^bcde^	3.81 ^h^	3.92 ^fgh^	4.18 ^abc^	4.20 ^abc^	3.99 ^defg^	4.10 ^bcde^	4.11 ^bcde^	4.25 ^ab^	3.95 ^efgh^	***
SD	0.01	0.07	0.06	0.01	0.15	0.04	0.01	0.01	0.19	0.05	0.02	0.06	0.04	0.02	0.02	0.17	
Intake of sugars, %	98.75 ^a^	98.33 ^ab^	98.16 ^ab^	98.51 ^ab^	98.27 ^ab^	98.55 ^ab^	98.52 ^ab^	98.74 ^a^	98.57 ^ab^	98.33 ^ab^	98.32 ^ab^	97.83 ^b^	98.71 ^a^	98.55 ^ab^	98.84 ^a^	96.34 ^c^	***
SD	0.02	0.02	0.05	0.00	0.01	0.00	0.01	0.02	0.01	0.01	0.01	0.00	0.00	0.01	0.03	2.66	
Fermentation yield (% of theoretical)	71.89 ^abcd^	75.91 ^ab^	75.77 ^ab^	76.59 ^a^	72.99 ^abc^	71.93 ^abcd^	74.26 ^abc^	69.68 ^cd^	75.62 ^ab^	76.07 ^ab^	74.99 ^ab^	74.24 ^abc^	71.69 ^abcd^	71.10 ^bcd^	76.01 ^ab^	67.25 ^d^	***
SD	5.16	1.98	13.20	3.09	2.23	0.59	5.93	32.03	0.65	2.29	6.88	1.61	0.00	0.19	3.65	15.99	

^1^ Signification codes: 0 < *** < 0.001 < ** < 0.01 < * < 0.05 < . < 0.1 < ° < 1. ^a–h^—mean values in rows denoted by different letters are significantly different (one-way ANOVA). SD—standard deviation.

**Table 5:** Major volatile compounds in fermented mashes obtained from rye cultivated with the use of different ashes as fertilisers.

Compound (mg/L)	Control	CC, t/ha			WCH, t/ha			BDL, t/ha			CC25 + WCH75, t/ha			CC50 + BDL50, t/ha			*p*-Values Signification Codes ^1^
		2	4	8	2	4	8	2	4	8	2	4	8	2	4	8	
Ethanal	3.28 ^bc^	1.97 ^cd^	2.59 ^bcd^	3.15 ^bcd^	3.62 ^b^	3.69 ^b^	3.03 ^bcd^	1.78 ^d^	3.00 ^bcd^	3.44 ^b^	2.49 ^bcd^	2.41 ^bcd^	2.80 ^bcd^	2.29 ^bcd^	2.42 ^bcd^	8.98 ^a^	***
SD	1.20	0.08	0.29	0.37	0.18	0.08	0.07	0.04	0.16	0.11	0.30	0.45	0.10	0.23	0.95	0.71	
Propan-2-one	0.14 ^a^	0.10 ^a^	0.14 ^a^	0.11 ^a^	0.13 ^a^	0.14 ^a^	0.12 ^a^	0.12 ^a^	0.13 ^a^	0.12 ^a^	0.12 ^a^	0.12 ^a^	0.12 ^a^	0.12 ^a^	0.11 ^a^	0.12 ^a^	°
SD	0.02	0.00	0.01	0.02	0.00	0.01	0.01	0.01	0.01	0.01	0.03	0.01	0.02	0.00	0.03	0.01	
Methanol	1.984 ^bc^	2.27 ^abc^	2.62 ^a^	2.11 ^abc^	2.19 ^abc^	2.25 ^abc^	2.03 ^abc^	2.06 ^abc^	1.959 ^bc^	2.33 ^ab^	2.11 ^abc^	2.22 ^abc^	1.70 ^c^	2.14 ^abc^	1.99 ^bc^	1.94 ^bc^	**
SD	0.04	0.13	0.20	0.31	0.04	0.20	0.04	0.11	0.18	0.09	0.33	0.34	0.16	0.07	0.32	0.15	
Propan-1-ol	7.72 ^ab^	6.76 ^ab^	8.53 ^a^	8.08 ^a^	7.87 ^ab^	8.07 ^a^	7.49 ^ab^	8.77 ^a^	7.47 ^ab^	8.85 ^a^	6.80 ^ab^	6.51 ^ab^	6.80 ^ab^	7.87 ^ab^	8.79 ^a^	4.97 ^b^	**
SD	0.48	1.63	0.23	1.32	0.14	0.19	0.57	0.30	0.58	0.84	0.86	0.87	0.51	0.65	2.41	1.26	
Hexanal	0.70 ^ab^	0.59 ^ab^	0.81 ^a^	0.70 ^ab^	0.65 ^ab^	0.76 ^a^	0.67 ^ab^	0.70 ^ab^	0.65 ^ab^	0.75 ^a^	0.70 ^ab^	0.58 ^ab^	0.67 ^ab^	0.72 ^ab^	0.77 ^a^	0.47 ^b^	*
SD	0.04	0.15	0.02	0.12	0.01	0.06	0.02	0.02	0.02	0.08	0.11	0.10	0.06	0.09	0.16	0.16	
Butan-1-ol	0.31 ^a^	0.31 ^a^	0.32 ^a^	0.33 ^a^	0.33 ^a^	0.35 ^a^	0.36 ^a^	0.30 ^a^	0.26 ^a^	0.35 ^a^	0.27 ^a^	0.29 ^a^	0.30 ^a^	0.27 ^a^	0.27 ^a^	0.25 ^a^	*
SD	0.06	0.02	0.06	0.05	0.04	0.07	0.01	0.01	0.01	0.01	0.03	0.04	0.03	0.01	0.01	0.03	
Ethyl ethanoate	3.81 ^abc^	3.30 ^abc^	2.76 ^abc^	3.08 ^abc^	2.51 ^bc^	2.69 ^abc^	2.74 ^abc^	3.96 ^a^	3.59 ^abc^	3.23 ^abc^	2.22 ^c^	2.93 ^abc^	2.83 ^abc^	2.73 ^abc^	3.36 ^abc^	2.31 ^c^	**
SD	0.54	0.15	0.58	0.02	0.29	0.34	0.07	0.79	0.18	0.45	0.05	0.72	0.80	0.31	0.72	0.16	
2-Methylpropan-1-ol	110.16 ^a^	110.54 ^a^	116.26 ^a^	122.85 ^a^	91.05 ^ab^	119.64 ^a^	108.94 ^a^	123.36 ^a^	108.35 ^ab^	114.32 ^a^	103.55 ^ab^	83.32 ^ab^	101.89 ^ab^	107.35 ^ab^	125.01 ^a^	65.02 ^b^	**
SD	7.71	5.00	1.66	3.61	17.37	11.11	7.27	14.49	4.81	11.41	8.73	18.83	7.80	8.20	20.25	37.91	
3-Methylbutan-1-ol	92.69 ^cde^	112.80 ^a^	100.74 ^abcd^	108.62 ^ab^	95.13 ^bcde^	101.64 ^abc^	102.05 ^abc^	102.49 ^abc^	86.93 ^de^	108.29 ^ab^	93.04 ^cde^	97.55 ^bcde^	85.99 ^e^	92.07 ^cde^	93.58 ^cde^	90.85 ^cde^	***
SD	5.79	5.10	6.66	1.16	2.45	2.28	0.82	10.59	1.46	5.05	5.02	5.85	3.84	2.13	2.92	2.17	
2-Methylbutan-1-ol	43.19 ^ab^	46.91 ^ab^	45.01 ^ab^	49.10 ^a^	38.99 ^bc^	47.37 ^ab^	45.45 ^ab^	49.31 ^a^	40.71 ^abc^	47.24 ^ab^	41.99 ^abc^	38.87 ^bc^	40.22 ^abc^	42.32 ^abc^	45.95 ^ab^	33.58 ^c^	***
SD	1.32	2.12	2.28	0.63	3.97	1.75	1.45	4.64	0.62	1.45	2.43	3.41	3.01	1.25	3.38	7.49	

^1^ Signification codes: 0 < *** < 0.001 < ** < 0.01 < * < 0.05 < . < 0.1 < ° < 1. ^a–e^—mean values in rows denoted by different letters are significantly different (one-way ANOVA). SD—standard deviation.

**Table 6:** Minor volatile compounds (esters) in fermented mashes obtained from rye cultivated with the use of different ashes as fertilisers.

Compound (μg/L)	Control	CC, t/ha			WCH, t/ha			BDL, t/ha			CC25 + WCH75, t/ha			CC50 + BDL50, t/ha			*p*-Values Signification Codes ^1^
		2	4	8	2	4	2	4	8	2	4	2	4	8	2	4	
Ethyl methanoate	157.2 ^a^	72.6 ^bc^	126.3 ^abc^	145.4 ^ab^	148.6 ^ab^	159.6 ^a^	136.5 ^abc^	73.9 ^bc^	131.0 ^abc^	108.4 ^abc^	97.9 ^abc^	95.9 ^abc^	106.7 ^abc^	137.8 ^abc^	92.6 ^abc^	58.0 ^c^	***
SD	39.2	8.5	12.0	57.7	6.9	35.1	15.2	5.4	23.6	16.0	11.2	31.7	21.3	35.8	34.9	4.6	
Ethyl 2-methylbutanoate	0.7 ^a^	0.6 ^a^	0.6 ^a^	0.6 ^a^	0.5 ^a^	0.6 ^a^	0.4 ^a^	0.7 ^a^	0.5 ^a^	0.5 ^a^	0.6 ^a^	0.6 ^a^	0.5 ^a^	0.6 ^a^	0.6 ^a^	0.4 ^a^	*
SD	0.0	0.0	0.2	0.1	0.1	0.1	0.0	0.1	0.0	0.0	0.2	0.1	0.0	0.1	0.2	0.1	
Ethyl propanoate	46.8 ^a^	39.7 ^ab^	33.9 ^ab^	41.6 ^ab^	29.2 ^b^	38.0 ^ab^	33.3 ^ab^	44.5 ^ab^	41.5 ^ab^	43.5 ^ab^	30.8 ^b^	40.1 ^ab^	35.5 ^ab^	38.6 ^ab^	36.7 ^ab^	32.3 ^ab^	**
SD	5.6	1.8	6.2	0.9	4.4	6.5	3.8	5.4	4.3	6.4	0.9	8.5	9.7	2.7	2.8	1.0	
Ethyl 2-methylpropanoate	6.0 ^ab^	5.4 ^abc^	4.6 ^abcd^	5.5 ^abc^	4.1 ^bcd^	4.9 ^abc^	4.3 ^bcd^	6.4 ^a^	6.1 ^ab^	5.2 ^abc^	4.1 ^bcd^	3.8 ^cd^	4.7 ^abc^	4.9 ^abc^	6.4 ^a^	2.5 ^d^	***
SD	0.4	0.2	0.6	0.2	0.8	1.1	0.2	0.7	0.7	0.6	0.2	0.2	1.3	0.1	0.9	1.1	
Isobutyl ethanoate	9.9 ^ab^	5.5 ^bc^	8.4 ^abc^	9.4 ^ab^	6.1 ^bc^	9.1 ^ab^	7.6 ^abc^	8.6 ^abc^	11.7 ^a^	8.3 ^abc^	6.0 ^bc^	5.0 ^bc^	8.3 ^abc^	7.4 ^abc^	10.0 ^ab^	3.6 ^c^	***
SD	0.7	0.2	1.2	0.1	2.0	1.8	0.7	1.1	0.8	1.8	0.6	0.9	2.5	0.6	4.1	2.8	
Ethyl butanoate	5.2 ^ab^	4.1 ^ab^	4.4 ^ab^	5.4 ^a^	3.7 ^abc^	4.7 ^ab^	4.1 ^ab^	5.0 ^ab^	5.4 ^a^	4.8 ^ab^	3.4 ^bc^	3.5 ^bc^	3.9 ^ab^	4.1 ^ab^	5.0 ^ab^	2.2 ^c^	***
SD	0.6	0.2	0.8	0.0	0.6	0.7	0.1	0.3	0.4	0.7	0.2	0.2	0.8	0.3	1.0	1.0	
Ethyl 3-methylbutanoate	0.2 ^b^	0.3 ^a^	0.2 ^b^	0.3 ^a^	0.2 ^b^	0.2 ^b^	0.2 ^b^	0.2 ^b^	0.2 ^b^	0.2 ^b^	0.2 ^b^	0.2 ^b^	0.2 ^b^	0.2 ^b^	0.2 ^b^	0.1 ^c^	***
SD	0.0	0.0	0.0	0.0	0.0	0.0	0.0	0.0	0.0	0.0	0.0	0.0	0.0	0.0	0.0	0.0	
3-Methylbutyl ethanoate	41.0 ^ab^	27.8 ^bc^	35.5 ^ab^	41.7 ^ab^	30.5 ^abc^	36.9 ^ab^	32.2 ^abc^	38.0 ^ab^	47.7 ^a^	36.0 ^ab^	25.2 ^bc^	27.4 ^bc^	32.9 ^abc^	32.8 ^abc^	41.9 ^ab^	16.2 ^c^	***
SD	8.9	1.3	7.5	0.8	3.5	6.3	1.4	3.7	2.0	6.9	2.4	4.3	9.2	2.0	13.3	6.8	
2-Methylbutyl ethanoate	9.8 ^abc^	5.1 ^cd^	8.2 ^abcd^	9.5 ^abc^	6.3 ^bcd^	9.0 ^abc^	7.4 ^abcd^	8.8 ^abc^	11.5 ^a^	8.1 ^abcd^	5.7 ^bcd^	5.4 ^bcd^	8.0 ^abcd^	7.5 ^abcd^	10.2 ^ab^	3.3 ^d^	***
SD	1.8	0.2	1.5	0.2	1.6	1.7	0.7	0.8	0.7	2.0	0.5	0.8	2.3	0.5	3.9	2.5	
Ethyl pentanoate	1.4 ^abc^	1.7 ^ab^	1.3 ^bcd^	1.7 ^a^	1.2 ^cd^	1.5 ^abc^	1.3 ^abcd^	1.5 ^abc^	1.3 ^bcd^	1.4 ^abc^	1.1 ^cde^	1.3 ^abcd^	0.9 ^de^	1.3 ^cd^	1.2 ^cd^	0.7 ^e^	***
SD	0.3	0.1	0.2	0.0	0.2	0.2	0.1	0.1	0.1	0.0	0.0	0.2	0.1	0.0	0.0	0.0	
Ethyl hexanoate	10.2 ^bc^	11.1 ^abc^	13.1 ^abc^	16.9 ^a^	11.5 ^abc^	14.2 ^ab^	15.1 ^ab^	11.8 ^abc^	10.8 ^bc^	14.4 ^ab^	10.1 ^bc^	14.6 ^ab^	7.7 ^c^	7.6 ^c^	9.4 ^bc^	13.4 ^abc^	***
SD	2.6	0.5	2.8	1.1	2.4	1.4	2.6	1.3	1.1	1.1	0.5	4.1	1.6	1.0	1.1	1.9	
Ethyl octanoate	1.3 ^cd^	1.2 ^cd^	1.9 ^cd^	4.4 ^a^	1.6 ^cd^	3.3 ^ab^	2.0 ^bcd^	1.3 ^cd^	1.3 ^cd^	2.5 ^bc^	2.2 ^bcd^	1.5 ^cd^	1.1 ^d^	1.0 ^d^	1.4 ^cd^	1.2 ^cd^	***
SD	0.1	0.1	0.2	0.3	0.4	0.4	0.2	0.1	0.2	1.0	0.2	0.7	0.3	0.0	0.2	0.9	
Ethyl nonanoate	0.1 ^e^	0.1 ^e^	0.1 ^e^	0.4 ^a^	0.1 ^e^	0.3 ^b^	0.2 ^c^	0.1 ^e^	0.1 ^e^	0.2 ^c^	0.1 ^e^	0.1 ^e^	0.1 ^e^	0.1 ^e^	0.1 ^e^	0.1 ^e^	***
SD	0.0	0.0	0.0	0.1	0.0	0.0	0.0	0.0	0.0	0.0	0.0	0.0	0.0	0.0	0.0	0.0	
Ethyl decanoate	1.0 ^cd^	1.0 ^cd^	1.1 ^cd^	2.6 ^a^	1.0 ^cd^	2.0 ^ab^	1.3 ^cd^	0.8 ^d^	0.9 ^d^	1.6 ^bc^	1.2 ^bc^	0.8 ^d^	0.8 ^d^	0.8 ^d^	0.8 ^d^	0.7 ^d^	***
SD	0.1	0.0	0.1	0.1	0.2	0.2	0.1	0.0	0.1	0.5	0.1	0.3	0.1	0.0	0.1	0.4	
Ethyl dodecanoate	0.2 ^e^	0.4 ^cd^	0.3 ^de^	0.8 ^a^	0.3 ^de^	0.6 ^b^	0.4 ^cd^	0.2 ^e^	0.2 ^e^	0.5 ^bc^	0.3 ^de^	0.3 ^de^	0.2 ^e^	0.2 ^e^	0.2 ^e^	0.2 ^e^	***
SD	0.0	0.0	0.0	0.1	0.0	0.0	0.0	0.0	0.0	0.1	0.0	0.0	0.0	0.0	0.0	0.1	

^1^ Signification codes: 0 < *** < 0.001 < ** < 0.01 < * < 0.05 < . < 0.1 < ° < 1. ^a–e^—mean values in rows denoted by different letters are significantly different (one-way ANOVA). SD—standard deviation.

**Table 7:** Minor volatile compounds (carbonyl compounds and alcohols) in fermented mashes obtained from rye cultivated with the use of different ashes as fertilisers.

Compound (μg/L)	Control	CC, t/ha			WCH, t/ha			BDL, t/ha			CC25 + WCH75, t/ha			CC50 + BDL50, t/ha			*p*-Values Signification Codes ^1^
		2	4	8	2	4	8	2	4	8	2	4	8	2	4	8	
2-Methylpropanal	39.9 ^bc^	23.7 ^c^	36.1 ^bc^	33.0 ^bc^	36.7 ^bc^	51.0 ^ab^	34.8 ^bc^	22.88 ^c^	36.6 ^bc^	45.1 ^abc^	38.2 ^bc^	29.6 ^bc^	40.3 ^bc^	29.7 ^bc^	25.5 ^bc^	70.5 ^a^	***
SD	3.8	1.0	1.5	7.6	2.4	1.9	2.1	1.1	0.9	4.1	1.4	9.1	2.4	3.4	1.2	2.9	
2-Methylbutanal	4.2 ^b^	2.5 ^b^	3.6 ^b^	3.9 ^b^	4.1 ^b^	5.8 ^ab^	3.7 ^b^	2.7 ^b^	3.8 ^b^	4.9 ^b^	4.0 ^b^	3.4 ^b^	3.9 ^b^	2.9 ^b^	3.1 ^b^	10.8 ^a^	***
SD	0.8	0.2	0.1	0.7	0.2	0.2	0.1	0.2	0.1	0.3	0.2	0.6	0.1	0.3	0.7	6.6	
3-Methylbutanal	9.1 ^b^	6.5 ^b^	9.2 ^b^	9.9 ^b^	9.2 ^b^	11.2 ^ab^	8.1 ^b^	5.5 ^b^	7.9 ^b^	11.7 ^ab^	9.1 ^b^	7.3 ^b^	8.0 ^b^	6.6 ^b^	5.7 ^b^	19.6 ^a^	**
SD	2.1	0.7	1.1	1.6	0.3	0.2	0.3	0.6	0.3	1.0	0.6	2.6	0.2	0.4	1.1	11.5	
Furan-2-carbaldehyde	16.7 ^bc^	22.9 ^abc^	21.6 ^abc^	27.5 ^a^	21.3 ^abc^	26.6 ^ab^	23.6 ^abc^	19.5 ^abc^	16.4 ^bc^	22.3 ^abc^	19.6 ^abc^	23.6 ^abc^	14.7 ^c^	17.0 ^bc^	19.0 ^abc^	22.8 ^abc^	**
SD	1.9	0.5	3.2	10.2	0.6	2.6	1.4	1.5	0.6	5.1	1.4	4.0	0.4	1.5	2.4	0.7	
Butane-2,3-dione	26.6 ^c^	55.4 ^c^	44.2 ^c^	33.9 ^c^	392.5 ^a^	36.1 ^c^	35.0 ^c^	36.9 ^c^	23.3 ^c^	40.3 ^c^	46.5 ^c^	52.5 ^c^	22.7 ^c^	29.7 ^c^	74.2 ^bc^	262.5 ^ab^	***
SD	1.7	6.0	7.5	2.7	3.3	2.2	3.9	1.2	0.6	1.3	9.3	9.9	0.6	5.5	4.4	16.7	
1,1-Diethoxyethane	13.1 ^abcd^	7.9 ^cd^	9.1 ^cd^	21.0 ^a^	12.1 ^bcd^	19.0 ^ab^	13.9 ^abcd^	6.8 ^cd^	15.0 ^abc^	19.1 ^ab^	6.2 ^d^	7.2 ^cd^	11.4 ^bcd^	7.8 ^cd^	9.3 ^cd^	9.1 ^cd^	***
SD	6.7	0.4	2.1	2.1	4.4	1.4	1.1	0.4	0.9	1.1	1.1	2.4	3.0	0.9	5.2	2.8	
Hexan-1-ol	80.1 ^abc^	93.2 ^abc^	86.5 ^abc^	107.2 ^ab^	64.5 ^c^	121.5 ^a^	93.5 ^abc^	71.3 ^bc^	73.2 ^bc^	85.4 ^abc^	89.6 ^abc^	75.7 ^bc^	75.2 ^bc^	110.4 ^ab^	78.1 ^bc^	100.5 ^abc^	***
SD	23.5	30.8	7.5	2.7	13.1	13.7	12.1	3.9	5.8	1.3	17.5	5.6	12.2	14.8	15.3	2.6	
Octan-1-ol	4.5 ^a^	4.1 ^a^	5.8 ^a^	5.9 ^a^	4.0 ^a^	6.1 ^a^	4.8 ^a^	5.5 ^a^	4.8 ^a^	5.5 ^a^	4.8 ^a^	3.7 ^a^	4.2 ^a^	4.2 ^a^	4.7 ^a^	4.7 ^a^	.
SD	0.3	1.1	1.5	0.1	0.7	0.5	0.7	1.4	0.3	0.7	1.5	0.3	0.4	0.8	1.6	1.3	

^1^ Signification codes: 0 < *** < 0.001 < ** < 0.01 < * < 0.05 < . < 0.1 < ° < 1. ^a–d^—mean values in rows denoted by different letters are significantly different (one-way ANOVA). SD—standard deviation.

**Table 8:** Soil fertilisation design.

No.	Treatment *	Rate of Ash/Ashes ** (Per 7 kg of Soil)	Rate of Ash/Ashes (t/ha)
1	Control	-	-
2	CC 2 t/ha	8 g CC	2
3	CC 4 t/ha	16 g CC	4
4	CC 8 t/ha	32 g CC	8
5	WCH 2 t/ha	8 g WCH	2
6	WCH 4 t/ha	16 g WCH	4
7	WCH 8 t/ha	32 g WCH	8
8	BDL 2 t/ha	8 g BDL	2
9	BDL 4 t/ha	16 g BDL	4
10	BDL 8 t/ha	32 g BDL	8
11	CC25 + WCH75 2 t/ha	8 g (CC 25% + WCH 75%)	2
12	CC25 + WCH75 4 t/ha	16 g (CC 25% + WCH 75%)	4
13	CC25 + WCH75 8 t/ha	32 g (CC 25% + WCH 75%)	8
14	CC50 + BDL50 2 t/ha	8 g (CC 50% + BDL 50%)	2
15	CC50 + BDL50 4 t/ha	16 g (CC 50% + BDL 50%)	4
16	CC50 + BDL50 8 t/ha	32 g (CC 50% + BDL 50%)	8

* CC—ash from corn cob combustion; WCH—ash from wood chips combustion; BDL—ash from forest biomass combustion in the presence of defecation lime. ** to each sample traditional fertilisers were added in the following rates: 1 g (per 7 kg of soil) of ammonium nitrate (AN) 34% N and 1 g (per 7 kg of soil) of ammonium phosphate (AP) 12% N 52% P_2_O_5_.

## Data Availability

The original contributions presented in this study are included in the article. Further inquiries can be directed to the corresponding author.

## Supplementary Materials

The following supporting information can be downloaded at: https://www.mdpi.com/article/10.3390/molecules30153251/s1, Table S1: Macro- and microelements content of rye grains; Figure S1: Correlation matrix representing Pearson correlation coefficients between volatile compounds in fermented mashes and minerals content in rye grains (values in bold correspond to correlations that were significant at α = 0.05); Figure S2: *p*-values of Pearson correlations between volatile compounds and minerals.

## Abbreviations

The following abbreviations are used in this manuscript:

CC	Corn Cob
WCH	Wood Chips
BDL	Biomass with Defecation Lime
