# Supplementary material for: Fermentation Efficiency and Profile of Volatile Compounds in Rye Grain Mashes from Crops Fertilised with Agrifood Waste Ashes
Source: Molecules. 2025 Aug 2;30(15):3251. doi: 10.3390/molecules30153251 (PMC12348167; doi:10.3390/molecules30153251)
Supplement: Supplementary file 1 [file molecules-30-03251-s001.zip › molecules-3784791-supplementary/molecules-3784791_Supplementary material_Figure S1.pdf]

Variables	Ethanal	Propan-2-one	Methanol	Propan-1-ol	Hexanal	Butan-1-ol	Ethyl ethanoate	2-Methylpropan-1-ol	3-Methylbutan-1-ol	2-Methylbutan-1-ol	Ethyl methanoate	Ethyl 2-methylbutanoate	Ethyl propanoate	Ethyl 2-methylpropanoate	Isobutyl ethanoate	Ethyl butanoate	Ethyl 3-methylbutanoate	3-Methylbutyl ethanoate	2-Methylbutyl ethanoate	Ethyl pentanoate	Ethyl hexanoate	Ethyl octanoate	Ethyl nonanoate	Ethyl decanoate	Ethyl dodecanoate	2-Methylpropanal	2-Methylbutanal	3-Methylbutanal	Butane-2,3-dione	Hexan-1-ol	Furan-2-carbaldehyde	Octan-1-ol	1,1-Diethoxyethane	Acetic acid	Ethyl alcohol	N	P	K	Na	Ca	Mg	Cu	Zn	Mn	Fe	Pb	Cd
Ethanal	1
Propan-2-one	0.126	1
Methanol	-0.214	0.214	1
Propan-1-ol	-0.596	0.187	0.372	1
Hexanal	-0.583	0.333	0.379	0.875	1
Butan-1-ol	-0.220	0.195	0.395	0.457	0.390	1
Ethyl ethanoate	-0.419	-0.051	-0.126	0.479	0.204	0.025	1
2-Methylpropan-1-ol	-0.681	-0.020	0.211	0.846	0.824	0.392	0.536	1
3-Methylbutan-1-ol	-0.240	-0.352	0.602	0.334	0.157	0.644	0.177	0.411	1
2-Methylbutan-1-ol	-0.606	-0.166	0.340	0.763	0.669	0.564	0.494	0.921	0.710	1
Ethyl methanoate	-0.215	0.596	0.136	0.418	0.500	0.502	-0.021	0.329	-0.055	0.199	1
Ethyl 2-methylbutanoate	-0.727	-0.008	0.242	0.464	0.501	-0.098	0.456	0.607	0.165	0.523	0.090	1
Ethyl propanoate	-0.306	-0.015	-0.031	0.363	0.165	0.089	0.855	0.442	0.278	0.478	0.076	0.385	1
Ethyl 2-methylpropanoate	-0.633	-0.071	-0.066	0.721	0.558	0.089	0.856	0.838	0.179	0.704	0.187	0.621	0.683	1
Isobutyl ethanoate	-0.416	0.309	-0.127	0.684	0.665	0.151	0.619	0.753	-0.102	0.515	0.517	0.341	0.503	0.812	1
Ethyl butanoate	-0.581	0.129	0.073	0.792	0.667	0.315	0.747	0.865	0.245	0.740	0.475	0.501	0.670	0.912	0.916	1
Ethyl 3-methylbutanoate	-0.496	-0.398	0.222	0.528	0.310	0.351	0.513	0.683	0.692	0.792	0.198	0.511	0.577	0.657	0.350	0.658	1
3-Methylbutyl ethanoate	-0.509	0.232	-0.038	0.743	0.638	0.197	0.697	0.768	0.018	0.568	0.525	0.415	0.575	0.863	0.969	0.965	0.493	1
2-Methylbutyl ethanoate	-0.436	0.301	-0.125	0.710	0.666	0.144	0.635	0.750	-0.104	0.515	0.525	0.368	0.515	0.823	0.995	0.924	0.374	0.981	1
Ethyl pentanoate	-0.578	-0.153	0.440	0.547	0.351	0.519	0.515	0.668	0.757	0.797	0.330	0.540	0.557	0.605	0.356	0.678	0.907	0.511	0.375	1
Ethyl hexanoate	0.246	0.020	0.412	0.040	-0.090	0.553	-0.091	-0.009	0.625	0.258	0.109	-0.278	0.082	-0.231	-0.113	0.044	0.221	-0.042	-0.104	0.370	1
Ethyl octanoate	0.004	0.020	0.281	0.297	0.335	0.550	-0.150	0.388	0.521	0.507	0.428	0.026	0.081	0.053	0.231	0.340	0.476	0.250	0.229	0.478	0.703	1
Ethyl nonanoate	0.023	-0.082	0.208	0.245	0.214	0.609	-0.118	0.344	0.578	0.499	0.438	-0.066	0.118	0.052	0.181	0.325	0.564	0.226	0.184	0.538	0.707	0.965	1
Ethyl decanoate	-0.039	0.032	0.282	0.327	0.354	0.630	-0.095	0.438	0.562	0.556	0.501	0.011	0.139	0.112	0.282	0.405	0.537	0.304	0.275	0.550	0.675	0.984	0.979	1
Ethyl dodecanoate	0.010	-0.121	0.299	0.196	0.163	0.645	-0.115	0.315	0.677	0.508	0.401	-0.067	0.154	0.026	0.097	0.285	0.603	0.154	0.091	0.600	0.708	0.925	0.979	0.958	1
2-Methylpropanal	0.901	0.371	-0.141	-0.499	-0.351	-0.057	-0.490	-0.562	-0.274	-0.527	-0.014	-0.706	-0.286	-0.619	-0.277	-0.490	-0.567	-0.423	-0.317	-0.587	0.222	0.130	0.105	0.106	0.097	1
2-Methylbutanal	0.978	0.188	-0.167	-0.575	-0.510	-0.192	-0.446	-0.637	-0.221	-0.556	-0.219	-0.695	-0.282	-0.637	-0.399	-0.572	-0.515	-0.514	-0.425	-0.580	0.267	0.068	0.059	0.020	0.049	0.951	1
3-Methylbutanal	0.947	0.201	-0.024	-0.516	-0.443	-0.062	-0.481	-0.592	-0.088	-0.483	-0.136	-0.686	-0.272	-0.653	-0.402	-0.532	-0.442	-0.503	-0.435	-0.484	0.371	0.200	0.184	0.160	0.191	0.950	0.970	1
Butane-2,3-dione	0.558	0.062	-0.006	-0.294	-0.448	-0.076	-0.431	-0.607	-0.167	-0.558	-0.085	-0.447	-0.577	-0.511	-0.513	-0.548	-0.334	-0.466	-0.486	-0.360	0.039	-0.165	-0.106	-0.203	-0.121	0.370	0.464	0.454	1
Hexan-1-ol	0.252	-0.035	0.197	-0.106	0.080	0.169	-0.375	0.115	0.303	0.222	0.217	-0.097	-0.026	-0.212	-0.098	-0.073	0.262	-0.162	-0.121	0.182	0.289	0.523	0.548	0.548	0.587	0.340	0.319	0.352	-0.236	1
Furan-2-carbaldehyde	0.219	-0.141	0.486	-0.009	-0.086	0.528	-0.283	0.015	0.721	0.305	0.065	-0.174	-0.092	-0.289	-0.288	-0.086	0.346	-0.209	-0.276	0.433	0.882	0.733	0.764	0.711	0.792	0.178	0.250	0.337	0.134	0.523	1
Octan-1-ol	0.039	0.289	0.363	0.510	0.553	0.406	0.101	0.572	0.409	0.612	0.241	0.108	0.204	0.260	0.452	0.463	0.309	0.392	0.431	0.331	0.514	0.718	0.613	0.700	0.560	0.200	0.141	0.218	-0.305	0.440	0.457	1
1,1-Diethoxyethane	0.133	0.205	0.026	0.331	0.272	0.608	0.107	0.331	0.296	0.358	0.603	-0.301	0.283	0.209	0.498	0.509	0.362	0.491	0.481	0.364	0.527	0.743	0.792	0.809	0.765	0.291	0.158	0.261	-0.111	0.328	0.419	0.559	1
Acetic acid	0.111	0.139	-0.293	0.319	0.248	-0.106	0.255	0.197	-0.341	-0.028	0.229	-0.057	-0.047	0.337	0.506	0.347	-0.095	0.464	0.506	-0.166	-0.201	-0.087	-0.138	-0.094	-0.239	0.005	0.002	0.002	0.199	-0.382	-0.381	0.039	0.184	1
Ethyl alcohol	-0.675	0.004	0.269	0.664	0.596	0.658	0.324	0.704	0.442	0.676	0.558	0.253	0.274	0.563	0.602	0.740	0.569	0.684	0.604	0.694	0.241	0.474	0.503	0.557	0.506	-0.520	-0.662	-0.563	-0.396	-0.048	0.200	0.277	0.573	0.091	1
N	0.624	-0.098	-0.036	-0.397	-0.265	-0.224	-0.479	-0.241	-0.025	-0.200	-0.163	-0.371	-0.413	-0.450	-0.210	-0.341	-0.202	-0.311	-0.244	-0.296	0.336	0.326	0.277	0.261	0.234	0.522	0.607	0.631	0.157	0.499	0.353	0.373	0.095	0.114	-0.414	1
P	0.139	0.351	0.178	0.098	0.158	0.092	-0.205	0.027	-0.023	0.053	0.176	-0.109	-0.061	-0.128	0.018	-0.047	-0.099	-0.027	0.038	-0.010	0.207	0.173	0.144	0.154	0.112	0.261	0.246	0.141	-0.046	0.470	0.334	0.309	0.119	-0.345	-0.031	0.004	1
K	0.806	-0.148	-0.434	-0.551	-0.490	-0.503	-0.337	-0.528	-0.394	-0.537	-0.433	-0.489	-0.319	-0.467	-0.343	-0.548	-0.449	-0.451	-0.346	-0.675	-0.055	-0.180	-0.169	-0.250	-0.227	0.621	0.768	0.656	0.357	0.233	-0.003	-0.067	-0.166	0.149	-0.765	0.627	0.087	1
Na	0.646	-0.194	-0.441	-0.544	-0.484	-0.493	-0.438	-0.552	-0.427	-0.589	-0.244	-0.280	-0.439	-0.456	-0.429	-0.570	-0.308	-0.474	-0.408	-0.551	-0.213	-0.152	-0.114	-0.219	-0.168	0.438	0.577	0.482	0.575	0.160	-0.017	-0.316	-0.221	0.152	-0.671	0.419	-0.015	0.835	1
Ca	0.638	-0.135	-0.300	-0.410	-0.350	-0.353	-0.273	-0.327	-0.208	-0.305	-0.272	-0.260	-0.206	-0.344	-0.309	-0.430	-0.173	-0.389	-0.306	-0.409	-0.086	-0.112	-0.059	-0.145	-0.090	0.453	0.596	0.498	0.249	0.465	0.076	0.012	-0.167	0.002	-0.684	0.569	0.138	0.888	0.793	1
Mg	0.781	-0.121	-0.446	-0.628	-0.537	-0.519	-0.386	-0.595	-0.453	-0.614	-0.400	-0.523	-0.347	-0.522	-0.409	-0.625	-0.504	-0.527	-0.420	-0.716	-0.192	-0.325	-0.285	-0.367	-0.312	0.619	0.739	0.620	0.346	0.271	-0.108	-0.208	-0.245	0.069	-0.800	0.557	0.097	0.965	0.817	0.896	1
Cu	-0.053	0.203	0.487	0.039	0.080	-0.014	0.004	0.171	0.246	0.178	0.249	0.287	0.002	0.058	0.065	0.139	0.235	0.087	0.045	0.404	0.156	0.120	0.086	0.150	0.149	-0.102	-0.080	-0.014	-0.093	0.376	0.214	0.246	-0.054	0.003	0.032	0.374	0.071	-0.197	-0.214	0.004	-0.151	1
Zn	0.032	0.374	0.595	0.227	0.206	0.039	0.005	0.152	0.145	0.107	0.121	0.088	-0.184	0.046	0.147	0.125	-0.039	0.160	0.141	0.184	0.207	-0.020	-0.083	-0.018	-0.069	-0.019	0.018	0.017	0.122	0.092	0.225	0.329	-0.064	0.124	0.076	0.252	0.321	-0.063	-0.200	0.001	-0.076	0.699	1
Mn	0.055	0.409	-0.180	0.127	0.122	-0.200	0.235	0.033	-0.430	-0.162	0.271	0.215	0.033	0.239	0.224	0.108	-0.126	0.195	0.244	-0.113	-0.534	-0.490	-0.475	-0.450	-0.495	-0.016	-0.012	-0.122	0.217	-0.122	-0.460	-0.256	-0.229	0.445	-0.211	-0.249	0.139	0.150	0.299	0.288	0.232	0.187	0.264	1
Fe	0.029	0.476	0.061	0.214	0.155	0.267	0.397	0.198	0.009	0.129	0.465	0.176	0.253	0.306	0.359	0.354	0.115	0.373	0.365	0.282	0.016	-0.089	-0.056	-0.014	-0.044	-0.010	-0.035	-0.054	0.046	-0.028	-0.029	0.038	0.179	0.396	0.148	-0.179	0.054	-0.072	-0.014	0.095	-0.049	0.360	0.406	0.753	1
Pb	-0.028	0.613	0.298	0.217	0.294	0.296	-0.064	0.087	-0.057	0.026	0.694	0.009	0.041	-0.068	0.220	0.172	-0.036	0.211	0.219	0.129	0.118	0.087	0.083	0.142	0.085	0.054	-0.066	0.013	-0.098	0.262	-0.014	0.185	0.194	0.149	0.115	0.112	0.098	-0.192	-0.238	0.021	-0.101	0.551	0.364	0.320	0.473	1
Cd	0.294	-0.361	-0.360	-0.398	-0.370	-0.533	-0.347	-0.485	-0.381	-0.475	-0.519	-0.416	-0.203	-0.411	-0.393	-0.512	-0.404	-0.442	-0.380	-0.618	-0.174	-0.255	-0.258	-0.336	-0.297	0.243	0.323	0.229	0.074	0.014	-0.174	-0.292	-0.291	-0.226	-0.483	0.151	0.189	0.476	0.341	0.253	0.490	-0.511	-0.424	-0.342	-0.756	-0.402	1

Figure S1. Correlation matrix representing Pearson correlation coefficients between volatile compounds in fermented mashes and minerals content in rye grains (values in bold correspond to correlations that were significant at $\alpha = 0.05$).
