# Supplementary material for: Fermentation Efficiency and Profile of Volatile Compounds in Rye Grain Mashes from Crops Fertilised with Agrifood Waste Ashes
Source: Molecules. 2025 Aug 2;30(15):3251. doi: 10.3390/molecules30153251 (PMC12348167; doi:10.3390/molecules30153251)
Supplement: Supplementary file 1 [file molecules-30-03251-s001.zip › molecules-3784791-supplementary/molecules-3784791_Supplementary material_Figure S2.pdf]

Variables	Ethanal	Propan-2-one	Methanol	Propan-1-ol	Hexanal	Butan-1-ol	Ethyl ethanoate	2-Methylpropan-1-ol	3-Methylbutan-1-ol	2-Methylbutan-1-ol	Ethyl methanoate	Ethyl 2-methylbutanoate	Ethyl propanoate	Ethyl 2-methylpropanoate	Isobutyl ethanoate	Ethyl butanoate	Ethyl 3-methylbutanoate	3-Methylbutyl ethanoate	2-Methylbutyl ethanoate	Ethyl pentanoate	Ethyl hexanoate	Ethyl octanoate	Ethyl nonanoate	Ethyl decanoate	Ethyl dodecanoate	2-Methylpropanal	2-Methylbutanal	3-Methylbutanal	Butane-2,3-dione	Hexan-1-ol	Furan-2-carbaldehyde	Octan-1-ol	1,1-Diethoxyethane	Acetic acid	Ethyl alcohol	N	P	K	Na	Ca	Mg	Cu	Zn	Mn	Fe	Pb	Cd
Ethanal	0
Propan-2-one	0.642	0
Methanol	0.425	0.426	0
Propan-1-ol	0.015	0.488	0.156	0
Hexanal	0.018	0.207	0.147	<0.0001	0
Butan-1-ol	0.412	0.470	0.130	0.075	0.135	0
Ethyl ethanoate	0.106	0.853	0.641	0.061	0.448	0.927	0
2-Methylpropan-1-ol	0.004	0.942	0.433	<0.0001	<0.0001	0.133	0.032	0
3-Methylbutan-1-ol	0.372	0.181	0.014	0.207	0.560	0.007	0.512	0.114	0
2-Methylbutan-1-ol	0.013	0.540	0.197	0.001	0.005	0.023	0.052	<0.0001	0.002	0
Ethyl methanoate	0.424	0.015	0.615	0.107	0.049	0.048	0.938	0.214	0.839	0.461	0
Ethyl 2-methylbutanoate	0.001	0.976	0.366	0.070	0.048	0.719	0.076	0.013	0.542	0.038	0.741	0
Ethyl propanoate	0.248	0.956	0.911	0.167	0.542	0.743	<0.0001	0.087	0.298	0.061	0.779	0.141	0
Ethyl 2-methylpropanoate	0.008	0.793	0.809	0.002	0.025	0.743	<0.0001	<0.0001	0.507	0.002	0.487	0.010	0.004	0
Isobutyl ethanoate	0.109	0.244	0.638	0.003	0.005	0.578	0.011	0.001	0.706	0.041	0.040	0.197	0.047	0.000	0
Ethyl butanoate	0.018	0.634	0.788	0.000	0.005	0.235	0.001	<0.0001	0.361	0.001	0.063	0.048	0.004	<0.0001	<0.0001	0
Ethyl 3-methylbutanoate	0.051	0.127	0.408	0.035	0.243	0.183	0.042	0.004	0.003	0.000	0.461	0.043	0.019	0.006	0.183	0.006	0
3-Methylbutyl ethanoate	0.044	0.388	0.890	0.001	0.008	0.464	0.003	0.001	0.946	0.022	0.037	0.110	0.020	<0.0001	<0.0001	<0.0001	0.052	0
2-Methylbutyl ethanoate	0.091	0.257	0.644	0.002	0.005	0.596	0.008	0.001	0.701	0.041	0.037	0.160	0.041	<0.0001	<0.0001	<0.0001	0.153	<0.0001	0
Ethyl pentanoate	0.019	0.571	0.088	0.028	0.183	0.039	0.041	0.005	0.001	0.000	0.211	0.031	0.025	0.013	0.177	0.004	<0.0001	0.043	0.153	0
Ethyl hexanoate	0.358	0.940	0.112	0.883	0.740	0.026	0.738	0.975	0.010	0.336	0.687	0.298	0.763	0.389	0.677	0.872	0.412	0.878	0.702	0.159	0
Ethyl octanoate	0.988	0.941	0.291	0.264	0.204	0.027	0.579	0.137	0.038	0.045	0.098	0.925	0.764	0.846	0.390	0.197	0.062	0.351	0.393	0.061	0.002	0
Ethyl nonanoate	0.933	0.764	0.439	0.360	0.425	0.012	0.664	0.192	0.019	0.049	0.090	0.808	0.663	0.850	0.501	0.220	0.023	0.400	0.496	0.032	0.002	<0.0001	0
Ethyl decanoate	0.887	0.906	0.290	0.217	0.179	0.009	0.726	0.090	0.023	0.025	0.048	0.968	0.608	0.680	0.289	0.120	0.032	0.252	0.303	0.027	0.004	<0.0001	<0.0001	0
Ethyl dodecanoate	0.971	0.656	0.261	0.466	0.547	0.007	0.671	0.235	0.004	0.044	0.124	0.806	0.570	0.925	0.720	0.285	0.013	0.570	0.736	0.014	0.002	<0.0001	<0.0001	<0.0001	0
2-Methylpropanal	<0.0001	0.157	0.603	0.049	0.182	0.834	0.054	0.023	0.304	0.036	0.959	0.002	0.283	0.011	0.300	0.054	0.022	0.102	0.231	0.017	0.408	0.632	0.700	0.697	0.720	0
2-Methylbutanal	<0.0001	0.485	0.538	0.020	0.043	0.476	0.083	0.008	0.412	0.025	0.415	0.003	0.290	0.008	0.126	0.021	0.041	0.042	0.100	0.018	0.317	0.802	0.827	0.941	0.858	<0.0001	0
3-Methylbutanal	<0.0001	0.455	0.929	0.041	0.085	0.820	0.059	0.016	0.745	0.058	0.615	0.003	0.307	0.006	0.122	0.034	0.087	0.047	0.092	0.057	0.157	0.457	0.496	0.555	0.478	<0.0001	<0.0001	0
Butane-2,3-dione	0.025	0.818	0.984	0.268	0.082	0.780	0.096	0.013	0.536	0.025	0.755	0.082	0.019	0.043	0.042	0.028	0.206	0.069	0.056	0.170	0.887	0.542	0.697	0.452	0.656	0.158	0.070	0.077	0
Hexan-1-ol	0.346	0.899	0.465	0.695	0.768	0.533	0.152	0.672	0.254	0.408	0.419	0.722	0.925	0.431	0.719	0.789	0.326	0.548	0.654	0.500	0.278	0.038	0.028	0.028	0.017	0.197	0.228	0.181	0.379	0
Furan-2-carbaldehyde	0.416	0.602	0.056	0.973	0.751	0.036	0.288	0.956	0.002	0.250	0.810	0.519	0.735	0.278	0.280	0.752	0.189	0.436	0.302	0.094	<0.0001	0.001	0.001	0.002	0.000	0.509	0.351	0.202	0.622	0.038	0
Octan-1-ol	0.887	0.277	0.166	0.043	0.026	0.119	0.711	0.021	0.116	0.012	0.369	0.690	0.449	0.331	0.079	0.071	0.244	0.133	0.096	0.210	0.042	0.002	0.011	0.003	0.024	0.458	0.603	0.417	0.251	0.088	0.075	0
1,1-Diethoxyethane	0.624	0.446	0.925	0.210	0.308	0.013	0.693	0.210	0.265	0.173	0.014	0.256	0.288	0.437	0.050	0.044	0.168	0.053	0.059	0.166	0.036	0.001	0.000	0.000	0.001	0.274	0.558	0.329	0.682	0.214	0.106	0.024	0
Acetic acid	0.684	0.608	0.271	0.228	0.355	0.695	0.340	0.464	0.197	0.919	0.393	0.833	0.864	0.202	0.046	0.188	0.726	0.070	0.045	0.539	0.454	0.748	0.611	0.730	0.372	0.985	0.993	0.995	0.461	0.144	0.146	0.887	0.496	0
Ethyl alcohol	0.004	0.989	0.314	0.005	0.015	0.006	0.221	0.002	0.086	0.004	0.025	0.345	0.305	0.023	0.014	0.001	0.021	0.003	0.013	0.003	0.368	0.063	0.047	0.025	0.046	0.039	0.005	0.023	0.128	0.861	0.457	0.299	0.020	0.738	0
N	0.010	0.719	0.893	0.127	0.321	0.405	0.061	0.369	0.927	0.457	0.546	0.157	0.112	0.080	0.435	0.197	0.453	0.240	0.362	0.265	0.203	0.218	0.298	0.328	0.382	0.038	0.013	0.009	0.561	0.049	0.180	0.155	0.727	0.675	0.111	0
P	0.609	0.182	0.509	0.717	0.559	0.735	0.447	0.921	0.932	0.845	0.515	0.689	0.823	0.636	0.948	0.864	0.716	0.922	0.889	0.970	0.442	0.522	0.595	0.569	0.680	0.329	0.358	0.602	0.866	0.067	0.207	0.244	0.661	0.191	0.909	0.988	0
K	0.000	0.586	0.093	0.027	0.054	0.047	0.201	0.036	0.131	0.032	0.094	0.055	0.229	0.068	0.194	0.028	0.081	0.079	0.189	0.004	0.839	0.505	0.532	0.350	0.398	0.010	0.001	0.006	0.175	0.385	0.992	0.806	0.540	0.582	0.001	0.009	0.748	0
Na	0.007	0.472	0.088	0.030	0.057	0.053	0.090	0.027	0.099	0.016	0.363	0.294	0.089	0.076	0.097	0.021	0.246	0.063	0.117	0.027	0.428	0.575	0.675	0.416	0.534	0.090	0.019	0.059	0.020	0.554	0.951	0.233	0.411	0.574	0.004	0.106	0.956	<0.0001	0
Ca	0.008	0.618	0.260	0.115	0.184	0.179	0.306	0.216	0.440	0.251	0.308	0.331	0.444	0.192	0.244	0.096	0.521	0.136	0.250	0.115	0.751	0.678	0.828	0.591	0.741	0.078	0.015	0.050	0.353	0.070	0.780	0.964	0.538	0.994	0.003	0.021	0.611	<0.0001	0.000	0
Mg	0.000	0.656	0.083	0.009	0.032	0.039	0.140	0.015	0.078	0.011	0.125	0.038	0.188	0.038	0.116	0.010	0.046	0.036	0.105	0.002	0.477	0.219	0.285	0.163	0.239	0.011	0.001	0.010	0.190	0.311	0.689	0.440	0.361	0.800	0.000	0.025	0.722	<0.0001	0.000	<0.0001	0
Cu	0.846	0.451	0.056	0.886	0.769	0.959	0.989	0.527	0.358	0.509	0.351	0.281	0.993	0.830	0.811	0.608	0.381	0.748	0.870	0.121	0.564	0.659	0.752	0.580	0.581	0.707	0.767	0.959	0.732	0.152	0.426	0.359	0.842	0.992	0.907	0.154	0.795	0.464	0.426	0.989	0.576	0
Zn	0.908	0.154	0.015	0.398	0.443	0.887	0.986	0.574	0.593	0.694	0.654	0.746	0.496	0.866	0.588	0.646	0.886	0.554	0.602	0.495	0.441	0.940	0.761	0.949	0.800	0.943	0.948	0.949	0.653	0.736	0.402	0.214	0.814	0.648	0.779	0.347	0.225	0.817	0.457	0.998	0.778	0.003	0
Mn	0.840	0.116	0.504	0.640	0.652	0.457	0.382	0.903	0.097	0.549	0.309	0.423	0.904	0.373	0.404	0.692	0.642	0.468	0.363	0.676	0.033	0.054	0.063	0.081	0.051	0.953	0.964	0.654	0.419	0.654	0.073	0.338	0.393	0.084	0.432	0.353	0.607	0.579	0.261	0.279	0.387	0.488	0.324	0
Fe	0.914	0.063	0.823	0.425	0.567	0.318	0.127	0.461	0.973	0.635	0.069	0.514	0.345	0.249	0.173	0.179	0.673	0.155	0.164	0.291	0.953	0.744	0.837	0.959	0.872	0.970	0.897	0.842	0.867	0.917	0.916	0.888	0.506	0.129	0.585	0.507	0.843	0.792	0.959	0.726	0.856	0.171	0.118	0.001	0
Pb	0.917	0.012	0.262	0.419	0.270	0.266	0.814	0.747	0.833	0.923	0.003	0.975	0.879	0.803	0.413	0.523	0.894	0.433	0.415	0.634	0.665	0.749	0.760	0.600	0.754	0.841	0.809	0.962	0.719	0.326	0.959	0.493	0.472	0.582	0.673	0.680	0.719	0.477	0.375	0.940	0.710	0.027	0.165	0.227	0.064	0
Cd	0.268	0.169	0.171	0.127	0.158	0.033	0.188	0.057	0.145	0.063	0.040	0.109	0.451	0.113	0.132	0.042	0.120	0.087	0.146	0.011	0.520	0.340	0.335	0.204	0.263	0.364	0.222	0.394	0.786	0.959	0.519	0.273	0.273	0.401	0.058	0.578	0.483	0.062	0.197	0.345	0.054	0.043	0.102	0.195	0.001	0.123	0

Figure S2. p-values of Pearson correlations between volatile compounds and minerals.
