# Supplementary material for: Fermentation Efficiency and Profile of Volatile Compounds in Rye Grain Mashes from Crops Fertilised with Agrifood Waste Ashes
Source: Molecules. 2025 Aug 2;30(15):3251. doi: 10.3390/molecules30153251 (PMC12348167; doi:10.3390/molecules30153251)
Supplement: Supplementary file 1 [file molecules-30-03251-s001.zip › molecules-3784791-supplementary/molecules-3784791_Supplementary material_Table S1.pdf]

Table S1. Macro- and microelements content of rye grains.

Fertiliser	Dose of ash (Mg/ha)	Nitrogen (g/kg)	Phosphorus (g/kg)	Potassium (g/kg)	Sodium (g/kg)	Calcium (g/kg)	Magnesium (g/kg)	Copper (mg/kg)	Zinc (mg/kg)	Manganese (mg/kg)	Iron (mg/kg)	Lead (mg/kg)	Cadmium (mg/kg)
Control	-	11.5^{a}	4.13^{ab}	3.42^{a}	0.14^{b}	0.36^{ab}	0.52^{ab}	5.64^{b}	17.4^{ab}	21.3^{e}	40.9^{d}	0.23^{b}	0.05^{a}
CC	2 t/ha	12.4^{ab}	3.93^{ab}	3.03^{a}	0.12^{b}	0.36^{ab}	0.51^{ab}	6.06^{c}	19.4^{b}	15.7^{b}	30.5^{bc}	0.16^{a}	0.07^{ab}
	4 t/ha	14.0^{c}	4.29^{ab}	3.61^{ab}	0.09^{ab}	0.37^{ab}	0.51^{ab}	6.35^{c}	23.6^{c}	16.5^{bc}	32.7^{c}	0.24^{b}	0.06^{ab}
	8 t/ha	14.9^{c}	3.93^{ab}	4.14^{bc}	0.17^{c}	0.42^{b}	0.50^{ab}	5.60^{b}	17.1^{ab}	12.8^{a}	28.0^{b}	0.20^{ab}	0.08^{b}
WCH	2 t/ha	11.9^{a}	4.28^{ab}	3.22^{a}	0.19^{cd}	0.31^{ab}	0.49^{ab}	5.10^{b}	19.3^{b}	18.2^{bc}	31.3^{bc}	0.20^{ab}	0.08^{b}
	4 t/ha	12.9^{b}	6.11^{c}	3.78^{b}	0.13^{b}	0.41^{b}	0.54^{ab}	5.51^{b}	20.4^{bc}	17.0^{c}	32.5^{c}	0.20^{ab}	0.08^{b}
	8 t/ha	13.8^{bc}	4.71^{ab}	3.69^{b}	0.09^{ab}	0.35^{ab}	0.55^{ab}	5.63^{b}	19.3^{b}	15.7^{b}	32.1^{bc}	0.23^{b}	0.09^{c}
BDL	2 t/ha	12.2^{ab}	4.60^{ab}	3.61^{ab}	0.09^{ab}	0.38^{ab}	0.49^{ab}	5.16^{b}	18.7^{b}	16.3^{bc}	28.2^{b}	0.18^{ab}	0.10^{c}
	4 t/ha	13.3^{b}	4.46^{ab}	3.35^{a}	0.08^{ab}	0.26^{a}	0.50^{ab}	5.96^{bc}	21.0^{c}	16.2^{bc}	29.7^{b}	0.20^{ab}	0.10^{c}
	8 t/ha	11.9^{a}	4.45^{ab}	2.88^{a}	0.05^{a}	0.24^{a}	0.44^{a}	4.47^{ab}	16.9^{ab}	12.9^{a}	24.8^{b}	0.18^{ab}	0.12^{c}
CC25 + WCH75	2 t/ha	13.9^{bc}	4.44^{ab}	3.34^{a}	0.14^{b}	0.28^{a}	0.49^{ab}	5.58^{b}	17.1^{ab}	14.0^{ab}	21.6^{a}	0.17^{ab}	0.12^{c}
	4 t/ha	11.4^{a}	4.88^{b}	3.45^{a}	0.13^{b}	0.30^{a}	0.50^{ab}	4.72^{ab}	17.6^{ab}	14.0^{ab}	26.6^{b}	0.18^{ab}	0.13^{d}
	8 t/ha	12.2^{ab}	3.83^{a}	4.92^{c}	0.18^{c}	0.42^{b}	0.67^{c}	3.66^{a}	14.3^{a}	15.4^{b}	25.8^{b}	0.17^{ab}	0.12^{c}
CC50 + BDL50	2 t/ha	12.8^{b}	4.84^{ab}	4.28^{bc}	0.17^{c}	0.48^{b}	0.66^{c}	5.86^{bc}	18.2^{ab}	18.4^{cd}	27.3^{b}	0.24^{b}	0.13^{d}
	4 t/ha	12.5^{ab}	4.75^{ab}	5.30^{c}	0.21^{d}	0.43^{b}	0.63^{b}	4.68^{ab}	19.4^{b}	19.1^{d}	31.5^{bc}	0.15^{a}	0.11^{c}
	8 t/ha	16.2^{d}	4.67^{ab}	8.70^{d}	0.30^{e}	0.65^{c}	0.99^{d}	5.29^{b}	19.0^{b}	16.6^{bc}	28.3^{b}	0.18^{ab}	0.14^{e}

$^{a-e}$ – differences between mean values in columns marked with different letters are statistically significant (ANOVA, $p<0.05$). CC – ash from corn cob combustion; WCH – ash from wood chips combustion; BDL – ash from forest biomass combustion in the presence of defecation lime.
